# A general method for quantifying ligand binding to unmodified receptors using *Gaussia* luciferase

**DOI:** 10.1016/j.jbc.2021.100366

**Published:** 2021-02-02

**Authors:** András Dávid Tóth, Dániel Garger, Susanne Prokop, Eszter Soltész-Katona, Péter Várnai, András Balla, Gábor Turu, László Hunyady

**Affiliations:** 1 Department of Physiology, Faculty of Medicine, Semmelweis University, Budapest, Hungary; 2 MTA-SE Laboratory of Molecular Physiology, Eötvös Loránd Research Network, Budapest, Hungary; 3 Department of Internal Medicine and Hematology, Semmelweis University, Budapest, Hungary; 4 Szentágothai János Doctoral School of Neuroscience, Semmelweis University, Budapest, Hungary

**Keywords:** bioluminescence resonance energy transfer (BRET), G protein–coupled receptor (GPCR), cell surface receptor, signal transduction, molecular pharmacology, *Gaussia* luciferase (GLuc), NanoLuciferase (NanoLuc), ligand binding, α_1A_AR, α_1A_ adrenergic receptor, β_2_AR, β_2_ adrenergic receptor, AT_1_R, AT_1_ angiotensin receptor, BRET, bioluminescence resonance energy transfer, D_1_R, D_1_ dopamine receptor, DN-Dyn, dominant negative form of dynamin2A, EGFR, epidermal growth factor receptor, GLuc, *Gaussia* luciferase, GPCR, G protein–coupled receptor, NanoLuc, NanoLuciferase, PIP_2_, phosphatidylinositol 4,5-bisphosphate, PM, plasma membrane, SRE, serum response element, TAMRA‒AngII, red fluorophore–conjugated angiotensin II, TfR, transferrin receptor

## Abstract

Reliable measurement of ligand binding to cell surface receptors is of outstanding biological and pharmacological importance. Resonance energy transfer–based assays are powerful approaches to achieve this goal, but the currently available methods are hindered by the necessity of receptor tagging, which can potentially alter ligand binding properties. Therefore, we developed a tag-free system to measure ligand‒receptor interactions in live cells using the *Gaussia* luciferase (GLuc) as a bioluminescence resonance energy transfer donor. GLuc is as small as the commonly applied Nanoluciferase but has enhanced brightness, and its proper substrate is the frequently used coelenterazine. In our assay, bystander bioluminescence resonance energy transfer is detected between a GLuc-based extracellular surface biosensor and fluorescent ligands bound to their unmodified receptors. The broad spectrum of applications includes equilibrium and kinetic ligand binding measurements for both labeled and competitive unlabeled ligands, and the assay can be utilized for different classes of plasma membrane receptors. Furthermore, the assay is suitable for high-throughput screening, as evidenced by the identification of novel α_1_ adrenergic receptor ligands. Our data demonstrate that GLuc-based biosensors provide a simple, sensitive, and cost-efficient platform for drug characterization and development.

The superfamily of plasma membrane (PM) receptors consists of a diverse range of signaling proteins, such as G protein–coupled receptors (GPCRs), tyrosine kinase receptors, enzyme-linked receptors, nutrient receptors, or ion channels. In addition to their important role in sensing environmental signals, their altered function is commonly reported in a wide array of pathological conditions. Accordingly, most of our currently prescribed drugs target cell surface receptors (1). A growing number of evidences indicates that characteristics of drug‒receptor interaction may profoundly shape the pharmacological outcome. In addition to affinity and efficacy, kinetic ligand parameters were also shown to be decisive factors for the clinical action of drugs. Association and dissociation rate constants (k_on_ and k_off_) of drugs, by defining ligand residence time, have been linked to various drug properties, such as duration of action, efficacy, or occurrence of side effects (2, 3, 4, 5, 6, 7). Therefore, the proper characterization of drug‒receptor interactions represents a major task for drug development. Although various techniques with different advantageous properties are available, they all have serious drawbacks (8). For example, traditional radioligand binding measurements are ponderous and burdensome, especially in case of kinetic binding measurements, and the strict rules required for handling radioactive compounds limit their use. In contrast, fluorescence-based approaches are not hindered by the practical limitations surrounding the use of radioactivity but often suffer from low signal-to-noise ratio due to the autofluorescence of biological samples. By overcoming these limitations, resonance energy transfer–based measurements have revolutionized the application of fluorescent ligands in binding measurements (9, 10, 11, 12, 13). In these assays, resonance energy transfer is detected between a fluorescent or luminescent donor attached to a receptor and a fluorophore conjugated to a receptor ligand. Since the efficiency of resonance energy transfer strongly depends on the molecular proximity, a condition that is met during ligand binding, the signal-to-noise ratio is greatly enhanced. Time-resolved Förster resonance energy transfer (FRET) and bioluminescence resonance energy transfer (BRET) have been successfully used to detect ligand binding of various receptors for equilibrium and kinetic measurements as well. However, these methods require the covalent labeling of the target protein, which may substantially alter the receptor function. Furthermore, the high cost of some substrates may limit their use in high-throughput applications. Therefore, there is a continuous need for new and surpassing assays to assess ligand‒receptor binding. Here we report a novel BRET-based approach to measure ligand binding of cell surface receptors. The method is cost-effective, does not need receptor modification, and is also applicable in high-throughput drug screenings.

## Results

### Gaussia luciferase is a small and bright luciferase

**Figure 1:**
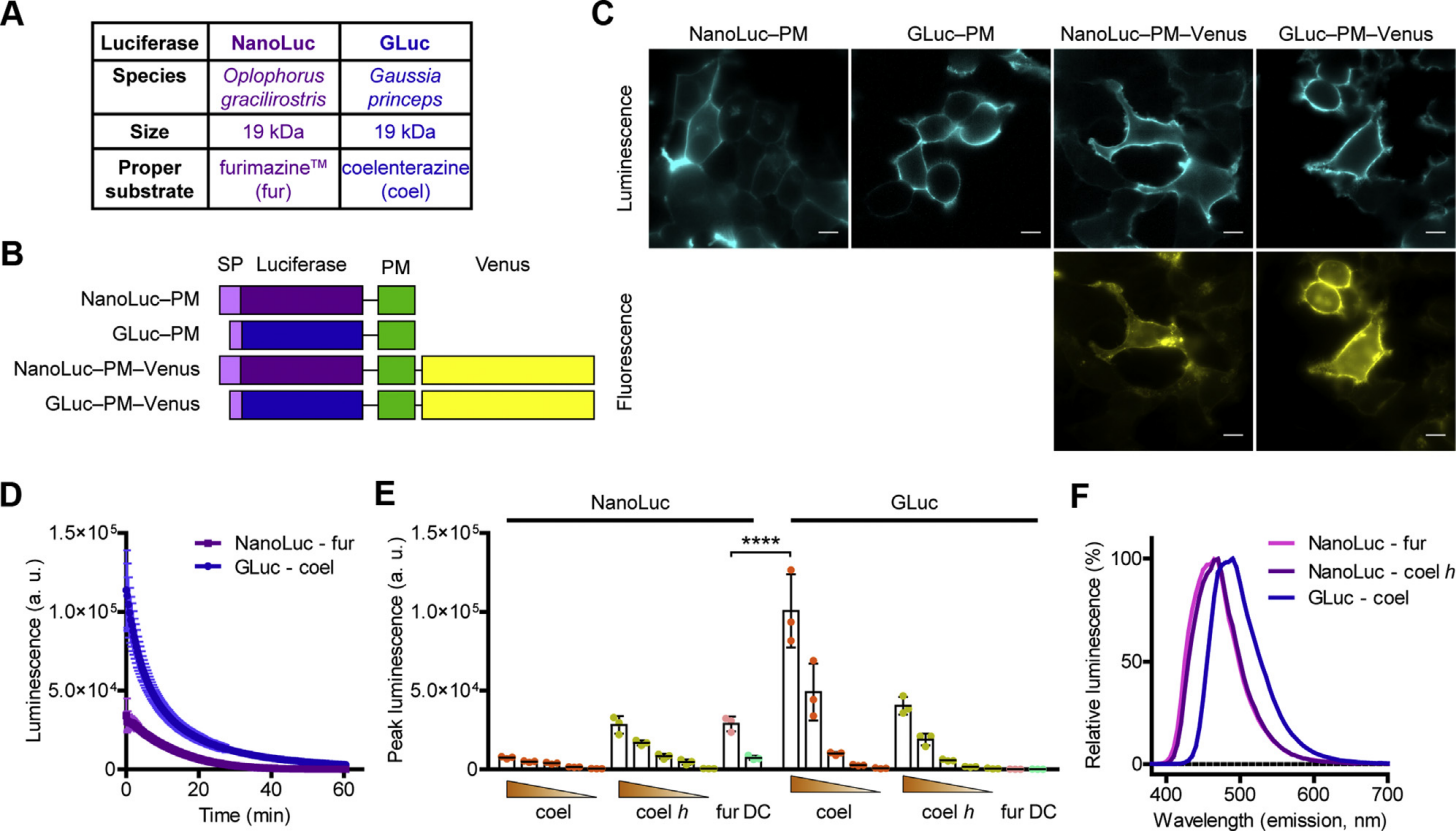
**GLuc is a bright alternative to NanoLuc.***A*, general characteristics of NanoLuc and GLuc. *B*, schematic structures of the used constructs. *C*, bioluminescent and fluorescent images show that the PM-targeted constructs have proper PM localization. Representative images from three independent experiments; the scale bars represent 10 μm. *D*, temporal decay of bioluminescence of NanoLuc and GLuc. Furimazine (fur; used in 1:200 dilution, stock concentration was not provided by Promega, the suggested dilution in protocol #TM439 is 1:500) or native coelenterazine (coel; 50 μM) was added as substrates, as indicated, and luminescence was continuously measured for 60 min, *n* = 3, data are mean ± SD. τ_NanoLuc_ = 12.2 min and τ_GLuc_ = 9.5 min assessed with one phase decay curve fitting. *E*, peak luminescence of GLuc and NanoLuc upon treatment with different substrates in distinct concentrations: native coelenterazine (coel; 50, 15, 5, 1.5, and 0.5 μM), coelenterazine *h* (coel *h*; 50, 15, 5, 1.5, and 0.5 μM), furimazine (fur; 1:200) and DeepBlueC (DBC; 5 μM). In *D* and *E*, the experiments were performed with the GLuc–PM–Venus and NanoLuc–PM–Venus constructs, and the measured luminescence was normalized to Venus fluorescence. Scatter dot plots with bars are shown, data are mean + SD. ∗∗∗∗*p* < 0.0001, one-way ANOVA, Tukey post hoc test, *n* = 3. *F*, emission spectra of GLuc and NanoLuc. GLuc–PM and NanoLuc–PM constructs were used for the measurements. Substrates were native coelenterazine (coel; 5 μM) for GLuc and coelenterazine *h* (coelenterazine *h*; 5 μM) or furimazine (fur; 1:200) for NanoLuc. Data are means of three independent experiments. GLuc, *Gaussia* luciferase; NanoLuc, NanoLuciferase; PM, plasma membrane.

Since N-terminal receptor tagging with naturally nonsecreted and high-molecular-weight luciferases (such as *Renilla* luciferase) induces endoplasmic retention and impaired PM expression of several receptors, it was not possible to study receptor‒ligand binding with BRET for a long time (8). This limitation has been overcome by the development of the small luciferase NanoLuciferase (NanoLuc) (14), a variant of the naturally secreted luciferase of the deep-sea shrimp *Oplophorus gracilirostris*, which has made BRET suitable to detect ligand binding of NanoLuc-tagged receptors. However, the high cost of furimazine, the proper substrate of NanoLuc, seriously hinders the widespread application, especially in high-throughput experiments. Therefore, we aimed to establish a system using an alternative coelenterazine-utilizing luciferase that is also secreted naturally; thus, it may not induce incorrect folding of proteins tagged on the extracellular side. The luciferase of the marine copepod *Gaussia princeps* (*Gaussia* luciferase, GLuc) has the same molecular weight as NanoLuc (19 kDa), but its appropriate substrate is a natural substance, the native coelenterazine (Fig. 1*A*) (15). Since GLuc is rapidly inactivated, we used a mutant of GLuc (GLucM23) (16), which emits light substantially longer and was shown to be even 10-fold brighter than the wildtype enzyme. First, we compared the properties of GLuc and NanoLuc with constructs that label the extracellular surface of the PM (GLuc‒PM and NanoLuc‒PM) by fusing them to a transmembrane domain. We also created versions of the constructs that were intracellularly tagged with Venus to perform expression-normalized comparisons (Fig. 1*B*). Using bioluminescent and fluorescent image acquisition, we verified that all constructs had proper PM localization (Fig. 1*C*). As shown in Figure 1, *D* and *E*, GLuc was found to have extreme brightness, was even brighter than NanoLuc, and both luciferases produced long-lasting (glow-type) luminescence (Fig. 1*D*). We tested the effect of different substrates in multiple concentrations. In agreement with previous studies, GLuc was the brightest when native coelenterazine was used (15, 16). The GLuc-emitted luminescence was already sufficiently detectable in the presence of 5 μM coelenterazine; therefore, this concentration was used in the experiments. Similar to previous reports (17, 18), we found that NanoLuc efficiently utilizes some other coelenterazine derivatives as well, such as 2-deoxycoelenterazine (coelenterazine *h*). Although coelenterazine *h* was shown to be inferior as a substrate of NanoLuc compared with furimazine, it still induced sufficient brightness to perform BRET measurements in our setup. We compared the emission spectra of GLuc and NanoLuc, and the emission of the former was right-shifted by approximately 20 nm (Fig. 1*F*), which may be beneficial for the excitation of red-emitting acceptors.

### GLuc is suitable for ligand binding applications

**Figure 2:**
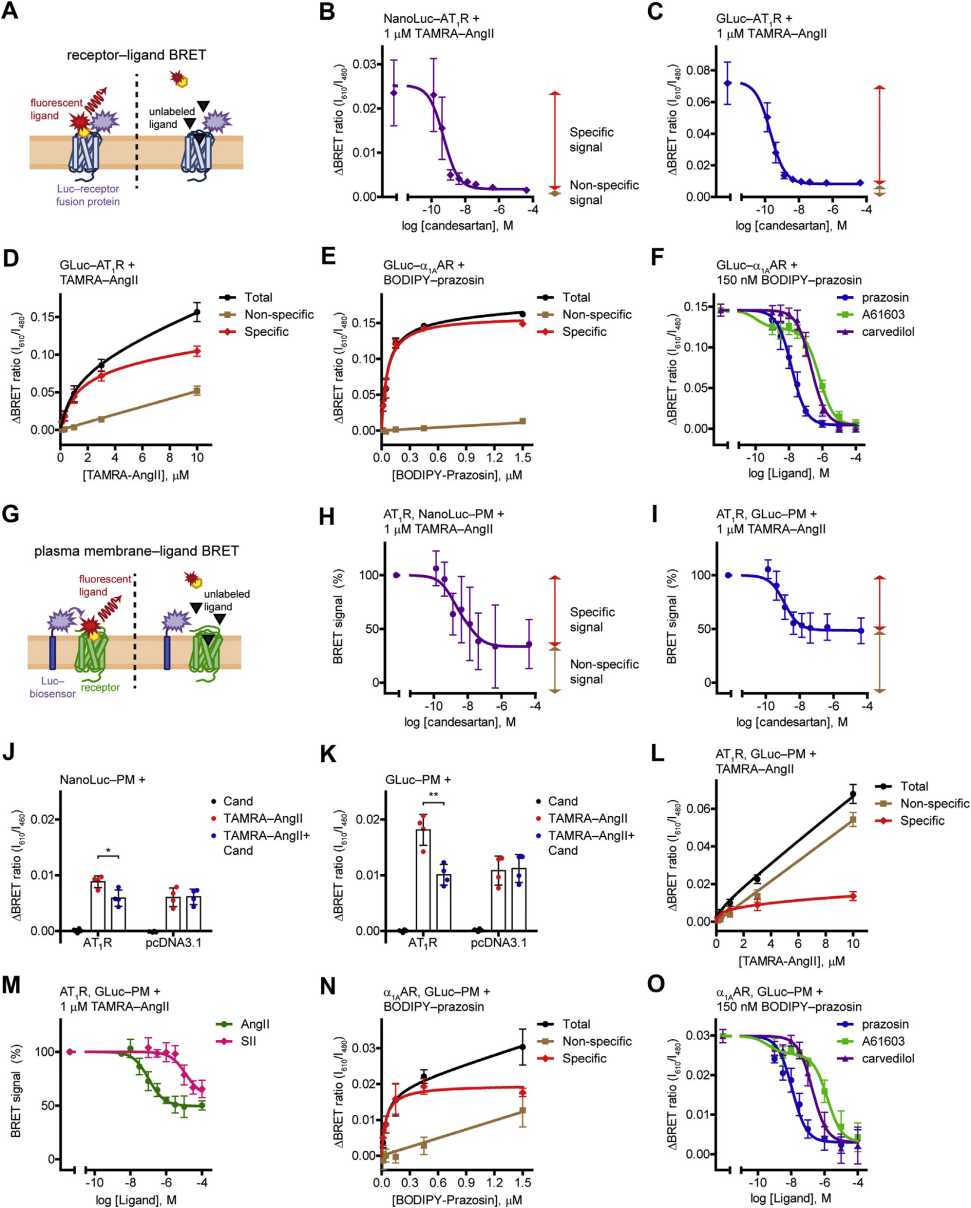
**Measurement of ligand binding of tagged or unmodified receptors in equilibrium.***A*, schematic representation of the receptor–ligand BRET measurements. Binding of a fluorescent ligand to its luciferase (Luc)-tagged receptor results in the molecular proximity of the BRET donor and acceptor molecules, which promotes resonance energy transfer and increase of the BRET signal. A competitor unlabeled ligand prevents the receptor binding of the fluorescent ligand, thus the specific BRET signal does not develop. *B* and *C*, competitive binding curves of NanoLuc–AT_1_R (*B*) and GLuc–AT_1_R (*C*). One micromolar TAMRA–AngII and increasing concentrations of candesartan were applied. *Red* and *brown two-headed arrow*s indicate the specific (candesartan-sensitive) and the nonspecific (candesartan-insensitive) signal, respectively. Two site competitive binding curves were fitted, *B*: IC_50_Hi_ = 520 pM, IC_50_Lo_ = 1 nM; *n* = 4. *C*: IC_50_Hi_ = 207 pM, IC_50_Lo_ = 1.3 nM; *n* = 3. *D*, concentration dependency of TAMRA–AngII binding to GLuc–AT_1_R. The BRET ratio was measured in GLuc–AT_1_R–expressing cells upon treatment of increasing concentrations of TAMRA–AngII. Nonspecific signal was assessed by cotreatment of 30 μM candesartan. Specific signal was calculated by subtracting the nonspecific signal from the total signal. Two site-specific, total, and nonspecific binding curves were fitted (K_D_Hi_ = 1.016 μM and K_D_Lo_ = 26.42 μM), *n* = 4. *E*, concentration dependency of BODIPY FL–prazosin binding to GLuc–ɑ_1A_AR. Total, specific, and nonspecific one site binding curves were fitted (K_D_ = 61.13 nM), *n* = 4. *F*, competitive binding curves of GLuc–ɑ_1A_AR. BODIPY FL–prazosin, 150 nM, was used as the labeled ligand; prazosin, A61603, and carvedilol were added in increasing concentrations. One site competitive binding curves were fitted on the points of the ɑ_1A_AR antagonists (prazosin, K_i_ = 4.17 nM and carvedilol, K_i_ = 62.7 nM); two site competitive binding curve was fitted on the points of the ɑ_1A_AR agonist A61603 (K_i_Hi_ = 32.5 pM, K_i_Lo_ = 187 nM), *n* = 6. *G*, schematic representation of the PM–ligand BRET measurements. Cells are cotransfected with untagged receptors and cell surface–targeted luciferase (Luc-biosensor). Fluorescent ligand binding to the receptor leads to its enrichment at the PM, which results in the increase of bystander BRET between the luciferase and the fluorescent ligand. Binding of unlabeled ligand to the receptor prevents the signal. *H* and *I*, competitive binding curves of untagged AT_1_R obtained with NanoLuc–PM (*H*) or GLuc–PM (*I*). Similar ligand treatments and curve fitting were used as in *B* and *C*; data are presented as the percentage of the ΔBRET ratio induced by TAMRA–AngII in the absence of candesartan. *H*: 100% = 0.0062 ± 0.0017, IC_50_Hi_ = 1.29 nM, IC_50_Lo_ = 19.6 nM; *n* = 4. *I*: 100% = 0.0168 ± 0.004, IC_50_Hi_ = 1.24 nM, IC_50_Lo_ = 248 μM; *n* = 4. *J* and *K*, BRET change upon TAMRA–AngII treatment with NanoLuc–PM (*J*) or GLuc–PM (*K*) in the presence or absence of receptor coexpression. Cells were cotransfected with AT_1_R or pcDNA3.1, 1 μM TAMRA–AngII, 30 μM candesartan, or vehicle treatments were applied, as indicated, *n* = 4. ∗*p* = 0.0173, ∗∗*p* = 0.0029, unpaired, two-tailed *t* test. *L*, concentration dependency of TAMRA–AngII binding to AT_1_R measured with GLuc–PM. Similar ligand treatments and curve fitting were used as in *D* (K_D_Hi_ = 287 nM and K_D_Lo_ = 18.46 μM), *n* = 4. *M*, AT_1_R binding of agonists with distinct functional effects assessed with GLuc–PM. Two site competitive binding curves were fitted, IC_50_Hi_ = 105 nM for AngII and IC_50_Hi_ = 1.28 μM for SII, IC_50_Lo_ fit values were ambiguous, *n* = 3. *N*, concentration dependency of BODIPY FL–prazosin binding to ɑ_1A_AR measured with GLuc–PM. Total, specific, and nonspecific one site binding curves were fitted (K_D_ = 46.63 nM), *n* = 4. *O*, competitive binding curves of prazosin, A61603, and carvedilol to ɑ_1A_AR assessed with GLuc–PM. Similar curve fitting was applied as in *F*. K_i_ = 2.72 nM was for prazosin, K_i_ = 43.8 nM was for carvedilol, K_i_Hi_ = 94.8 pM and K_i_Lo_ = 348.8 nM was for A61603, *n* = 4. Scatter dot plots with mean + SD are shown in *J* and *K*, and data are mean ± SD in the other panels. Measurements were performed after 2-h incubation with the indicated compounds at room temperature. BRET, bioluminescence resonance energy transfer; GLuc, *Gaussia* luciferase; PM, plasma membrane; TAMRA‒AngII, red fluorophore–conjugated angiotensin II.

To test the performance of GLuc in BRET assays, we measured receptor‒ligand BRET in a similar manner as it was described in a previous study with NanoLuc (Fig. 2*A*) (12). We expressed GLuc- or NanoLuc-tagged AT_1_ angiotensin receptor (AT_1_R) in HEK 293T cells as BRET donors and treated them with red fluorophore–conjugated angiotensin II (TAMRA‒AngII) as BRET acceptor for 2 h at room temperature to reach equilibrium binding (Fig. 2, *B* and *C*). In both cases, the binding of TAMRA‒AngII to the tagged receptors resulted in an increase of the BRET signal, as the molecular proximity between the donor and the acceptor caused resonance energy transfer. The increase of the BRET ratio was higher with GLuc‒AT_1_R, for which a possible explanation could be the greater overlap between the excitation spectrum of TAMRA and the emission spectrum of GLuc than that of NanoLuc. The specificity of the signal was verified by competitive ligand binding measurements. The competitive AT_1_R antagonist candesartan prevented a great portion of the BRET signal, proving that this part of the signal originated from specific interaction between TAMRA‒AngII and AT_1_R. The remaining nonspecific signal reflects random collisions between the donor and acceptor molecules (bystander BRET), whose amplitude is linearly proportional to the acceptor concentration. Accordingly, increasing concentrations of TAMRA‒AngII elevated the nonspecific signal linearly, whereas the specific binding was saturable (Fig. 2*D*). To avoid possible receptor internalization–related changes in the signal, we always overexpressed an internalization inhibitor protein, the dominant negative form of dynamin2A (DN-Dyn). We confirmed that its application does not disturb the ligand binding properties of the receptor (Fig. S1, *A* and *B*). We also tested GLuc in receptor‒ligand BRET measurements for another GPCR, the α_1A_ adrenergic receptor (α_1A_AR) (Fig. 2, *E* and *F*). The green-emitting fluorescent BODIPY FL‒prazosin was applied as the labeled ligand, the α_1A_AR antagonist prazosin and carvedilol or the α_1A_AR agonist A61603 were used as unlabeled ligands. Again, the ligand binding of the tagged receptor could be detected with GLuc. The tracer ligand was displaced by all α_1A_AR ligands, representing that binding of any orthosteric ligand can be measured.

### NanoLuc‒PM and GLuc‒PM biosensors allow the detection of ligand binding of unmodified GPCRs

It must be emphasized that the phenomenon of bystander BRET not only denotes a background signal but can also be beneficially exploited. Bystander BRET measurements are widely used to detect enrichment of a protein in a particular compartment, where a compartment-targeted molecule and the protein of interest are labeled with BRET partners. The labeling can even be indirect by tagging an interaction partner of the protein, which has the advantage that no molecular modification of the protein under investigation is required. Using bystander BRET, cellular redistribution of proteins, such as receptor internalization and β-arrestin recruitment, or lipid levels in the PM were previously successfully monitored (19, 20, 21, 22, 23). In these experiments, the acceptor accumulation in the donor’s proximity results in the elevation of the BRET signal. We applied a strategy based on similar principles to assess ligand binding of unmodified receptors. Instead of measuring BRET between luciferase-tagged receptors and their fluorescent ligands (“receptor‒ligand BRET”), we detected bystander BRET between luciferase-labeled PM and fluorescent ligands bound to their receptors (“PM‒ligand BRET”) (Fig. 2*G*). We coexpressed untagged AT_1_R with GLuc‒PM or NanoLuc‒PM constructs that label the extracellular side of the PM (Fig. 2, *H* and *I*). Treatment of cells with TAMRA‒AngII induced an increase of the BRET signal between the PM-targeted donor and TAMRA‒AngII. This signal originated from two types of bystander BRET. A significant portion of the signal could be prevented by the AT_1_R antagonist candesartan, showing that this signal reflects the specific interaction between TAMRA‒AngII and untagged AT_1_R, which caused the enrichment of TAMRA‒AngII at the PM. Accordingly, this signal was absent when no receptor was expressed (Fig. 2, *J* and *K*). The candesartan-insensitive part of the signal was caused by the other (nonspecific) type of bystander BRET (Fig. 2*L*), which is due to random collisions between PM–tagged luciferases and unbound acceptor-conjugated ligands. This candesartan-insensitive signal is similar and equally high as in receptor‒ligand BRET measurement (see Fig. 2, *D* and *L*). PM‒ligand BRET, in comparison with receptor‒ligand BRET, showed lower amplitude of the specific signal (ΔBRET values of the different setups are shown in Fig. 2, *B*, *C*, *J*, and *K*). This was in agreement with the fact that resonance energy transfer is greatly sensitive to the distance between donor and acceptor molecules, which is larger when the PM is labeled and not the target receptor. Remarkably, the half-maximal inhibitory concentration of candesartan for the high-affinity binding site (IC_50_Hi_) was significantly lower in the case of the receptor‒ligand BRET than that of the PM‒ligand BRET (Fig. S1, *C* and *D*). Accordingly, the dissociation constant of TAMRA‒AngII for the high-affinity binding site (K_D_Hi_) of the untagged receptor was smaller than the K_D_Hi_ for the GLuc-labeled receptor (Fig. 2, *D* and *L*), demonstrating that receptor tagging altered the ligand binding properties of the receptor. These results are in good agreement with previous studies showing altered ligand binding properties of AT_1_R upon N-terminal modification (24, 25) and suggest that, although receptor‒ligand BRET is a sensitive approach to detect ligand binding, the results obtained with this method should be interpreted cautiously. Since we achieved larger BRET signal changes with GLuc than with NanoLuc in our system (compare Fig. 2, *H* with *I* and Fig. 2, *J* with *K*), only GLuc BRET measurements were performed in the further experiments. In principle, a ligand binding assay should be able to detect the binding of any orthosteric ligands regardless of their functional effects. In accordance, we were able to determine the binding of both the high-affinity full agonist angiotensin II (AngII) and the low-affinity β-arrestin‒biased agonist [Sar^1^,Ile^4^,Ile^8^]-angiotensin II (SII) (26) with our assay (Fig. 2*M*).

We also measured the ligand binding of α_1A_AR with the PM‒ligand BRET setup (Fig. 2, *N* and *O*). With this approach, we were able to measure the competition binding of all three unlabeled ligands, and no significant shift of competition binding curves was observed compared with GLuc‒α_1A_AR, suggesting that the effect of N-terminal receptor tagging on receptor conformation may vary between GPCRs.

We tested if the amplitude of the specific signal correlates with the amount of the receptor DNA transfected (Fig. S2). We found a positive correlation for both AT_1_R and α_1A_AR, suggesting that a high level of receptor expression is advantageous for good signal-to-noise separation.

### PM‒ligand BRET using GLuc‒PM as a general approach for ligand binding measurement of multiple families of cell surface receptors

Theoretically, our PM‒ligand BRET system could be utilized for ligand binding detection of any cell surface receptors, if an appropriate fluorescent ligand is available. The approach is quick and easy, as it requires only the coexpression of the GLuc‒PM extracellular surface biosensor and the unmodified receptor, and no further protein fusion procedure is needed.

**Figure 3:**
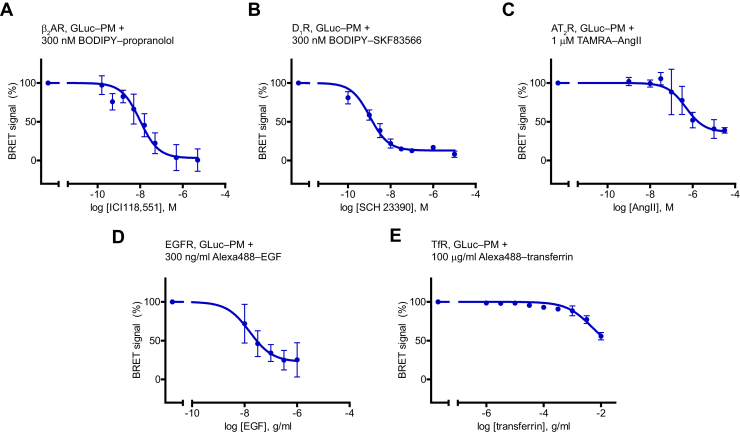
**Application of GLuc–PM to assess ligand binding of multiple cell surface receptor classes.** Competitive binding curves of ICI118,551 to β_2_AR (*A*, *n* = 5), of SCH 23390 to D_1_R (*B*, *n* = 3), of angiotensin II (AngII) to AT_2_R (*C*, *n* = 5), of epidermal growth factor (EGF) to EGFR (*D*, *n* = 4), and of transferrin to TfR (*E*, *n* = 3). In *A*–*E*, cells were cotransfected GLuc–PM with the examined receptor, and the indicated labeled ligands were used (300 nM BODIPY FL‒(S)-propranolol, 300 nM BODIPY FL‒SKF83566, 1 μM TAMRA‒AngII, 300 ng/ml biotinylated EGF conjugated to Alexa Fluor 488 streptavidin, or 100 μg/ml Alexa Fluor 488–conjugated human transferrin). Two-hour incubation was made on ice for β_2_AR and D_1_R, and at room temperature for the other receptors. For TfR, the BRET ratio was normalized to treatment of transferrin without fluorescent ligand. Data are mean ± SD, expressed as the percentage of the ΔBRET ratio induced by the treatment with fluorescent ligand without unlabeled ligand (0.0142 ± 0.07 (*A*), 0.0262 ± 0.0023 (*B*), 0.0119 ± 0.0016 (*C*), 0.0186 ± 0.0057 (*D*), and 0.6519 ± 0.0344 (*E*) values were 100%). One site competitive binding curves were fitted. IC_50_ values were 9.84 nM (*A*), 1.07 nM (*B*), 479 nM (*C*), 15.8 pg/l (*D*), and 4.54 μg/l (*E*). β_2_AR, β_2_ adrenergic receptor; AT_2_R, AT_2_ angiotensin receptor; GLuc, *Gaussia* luciferase; PM, plasma membrane; TAMRA‒AngII, red fluorophore–conjugated angiotensin II.

First, we adjusted our system to other GPCRs. We could detect the binding of BODIPY FL‒propranolol to the β_2_ adrenergic receptor (β_2_AR). Of interest, we found a consistent increase of the BRET ratio at low concentrations of the β_2_AR inverse agonist ICI118,552 instead of the expected drop in the signal (Fig. S3). We speculated that the 2-h incubation with the drug at room temperature may induce cell responses, for example, receptor externalization, which could influence the ligand binding results; therefore, we performed incubations on ice. Accordingly, we got regular competition binding curves (Fig. 3*A*). We could also successfully examine the ligand binding of the D_1_ dopamine receptor (D_1_R) or the AT_2_ angiotensin receptor (AT_2_R) (Fig. 3, *B* and *C*). A study of the latter with our assay could be especially useful in drug screening applications, because AT_2_R cannot be investigated with receptor signaling assays, as it does not signal in heterologous expression systems (27). It is interesting that AT_2_R had a TAMRA‒AngII affinity approximately two orders of magnitude higher than that of AT_1_R (Fig. S4), which explains the observed relatively higher IC_50_ of AngII to AT_2_R.

We also performed experiments with non-GPCR cell surface receptors. We examined the epidermal growth factor receptor (EGFR), a prototypical tyrosine kinase receptor (Fig. 3*D*), and the transferrin receptor (TfR), as an example of cell surface nutrient receptors (Fig. 3*E*). In both cases, we could detect the specific binding of the Alexa488-conjugated ligands to their receptors, which was prevented by unlabeled ligands. These results show that PM‒ligand BRET using GLuc is a versatile tool for ligand binding measurement of cell surface receptors.

### Real-time kinetic detection of ligand binding using GLuc BRET

**Figure 4:**
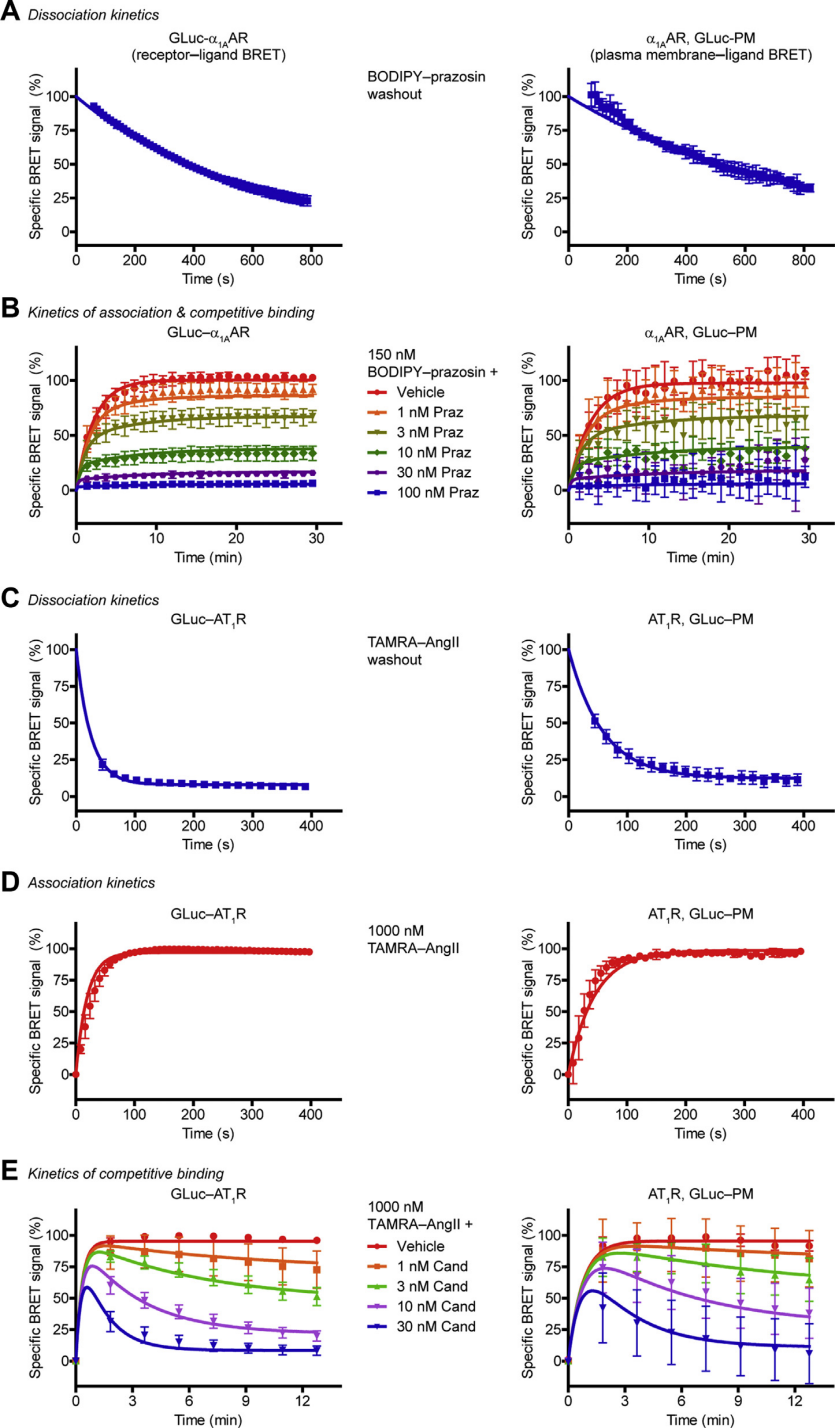
**Measurement of kinetic ligand parameters using GLuc.***Left panels*, kinetic receptor–ligand BRET measurements; *right panels*, kinetic PM–ligand BRET measurements. *A*, dissociation kinetics of BODIPY FL–prazosin. After 15-min treatment with 150 nM BODIPY FL–prazosin, the ligand was washed out, and the decline of the BRET ratio was followed. Prazosin was added to prevent rebinding. One phase exponential decay curves were fitted, k_off_ of BODIPY FL–prazosin was 1.84 × 10^−3^ s^−1^ for GLuc–ɑ_1A_AR, and k_off_ was 1.33 × 10^−3^ s^−1^ for ɑ_1A_AR assessed with GLuc–PM, *n* = 3. *B*, kinetics of association and competitive ligand binding to ɑ_1A_AR. Cells were treated with 150 nM BODIPY FL–prazosin and increasing concentrations of prazosin (Praz) simultaneously. k_on_ of BODIPY FL–prazosin was 3.86 × 10^4^ s^−1^M^−1^ for GLuc–ɑ_1A_AR, and k_on_ was 3.22 × 10^4^ s^−1^ for ɑ_1A_AR assessed with GLuc–PM. Prazosin k_on_ and k_off_ values were 2.38 × 10^6^ s^−1^M^−1^ and 3.51 × 10^−3^ s^−1^ for GLuc–ɑ_1A_AR, while they were 1.7 × 10^6^ s^−1^M^−1^ and 2.55 × 10^−3^ s^−1^, respectively, for ɑ_1A_AR determined with GLuc–PM, *n* = 5. *C*, dissociation kinetics of TAMRA–AngII. Cells were treated with 1 μM TAMRA–AngII for 15 min, then TAMRA–AngII was washed out and BRET was measured in real time. Candesartan was applied to prevent rebinding. One phase exponential decay curves were fitted, k_off_ of TAMRA–AngII was 0.04046 s^−1^ for GLuc–AT_1_R, and k_off_ was 0.01731 s^−1^ for AT_1_R assessed with GLuc–PM, *n* = 3. *D*, association kinetics of TAMRA–AngII (1 μM). One phase association curves were fitted to calculate k_on_, k_on_ of TAMRA–AngII was 1.15 × 10^4^ s^−1^M^−1^ for GLuc–AT_1_R, and k_on_ was 5.27 × 10^3^ s^−1^M^−1^ for AT_1_R measured with GLuc–PM, *n* = 3. *E*, kinetics of competitive binding to AT_1_R. Cells were simultaneously treated with 1 μM TAMRA–AngII and candesartan (Cand) in the indicated concentrations, and BRET was followed in real time. Candesartan k_on_ and k_off_ values were 5.4 × 10^5^ s^−1^M^−1^ and 1.23 × 10^−3^ s^−1^ for GLuc–AT_1_R, while they were 2.78 × 10^5^ s^−1^M^−1^ and 8.33 × 10^−4^ s^−1^, respectively, for AT_1_R assessed with GLuc–PM, *n* = 5. Data are mean ± SD. α_1A_AR, α_1A_ adrenergic receptor; BRET, bioluminescence resonance energy transfer; GLuc, *Gaussia* luciferase; PM, plasma membrane; TAMRA‒AngII, red fluorophore–conjugated angiotensin II.

A major advantage of resonance energy transfer–based ligand binding assays is the ability to detect ligand binding in real time and high temporal resolution, allowing simple determination of kinetic ligand parameters. We performed kinetic experiments on α_1A_AR in live cells with both GLuc BRET setups (receptor‒and PM‒ligand BRET). We measured the dissociation rate constant (k_off_) of the fluorescent ligand after its washout: the medium was replaced and supplemented with unlabeled ligand to prevent rebinding (Fig. 4*A*). We could follow the ligand dissociation in both setups. Next, we monitored the association of the fluorescent ligand to the receptor with real-time measurements (Fig. 4*B*) and determined the association rate constant (k_on_). Kinetic ligand parameters of unlabeled ligands can be assessed with the help of the Motulsky–Mahan equation (28). The k_off_ and k_on_ values of the unlabeled ligand can be calculated by measuring the ligand binding after simultaneous treatment of the labeled ligand and the unlabeled ligand in different concentrations. With the GLuc BRET system we could successfully determine these parameters of prazosin for α_1A_AR (Fig. 4*B*).

We also performed similar studies with AT_1_R. TAMRA‒AngII binds AT_1_R with two different affinities; however, the proportion of high- and low-affinity states shows a temporal change upon agonist binding because of the ternary complex formation with effectors. For the sake of simplicity, we fitted one site binding curves on the measured points. The k_off_ values of TAMRA‒AngII differed between receptor‒ligand BRET and PM‒ligand BRET setups (Fig. 4*C*), in agreement with the observation that the N-terminal tagging of AT_1_R alters its binding properties. Thereafter, we assessed the k_on_ values of TAMRA‒AngII (Fig. 4*D*) and calculated k_on_ and k_off_ for the unlabeled ligand candesartan in kinetic competitive ligand binding measurements (Fig. 4*E*).

### Screening of receptor ligands with GLuc BRET

**Figure 5:**
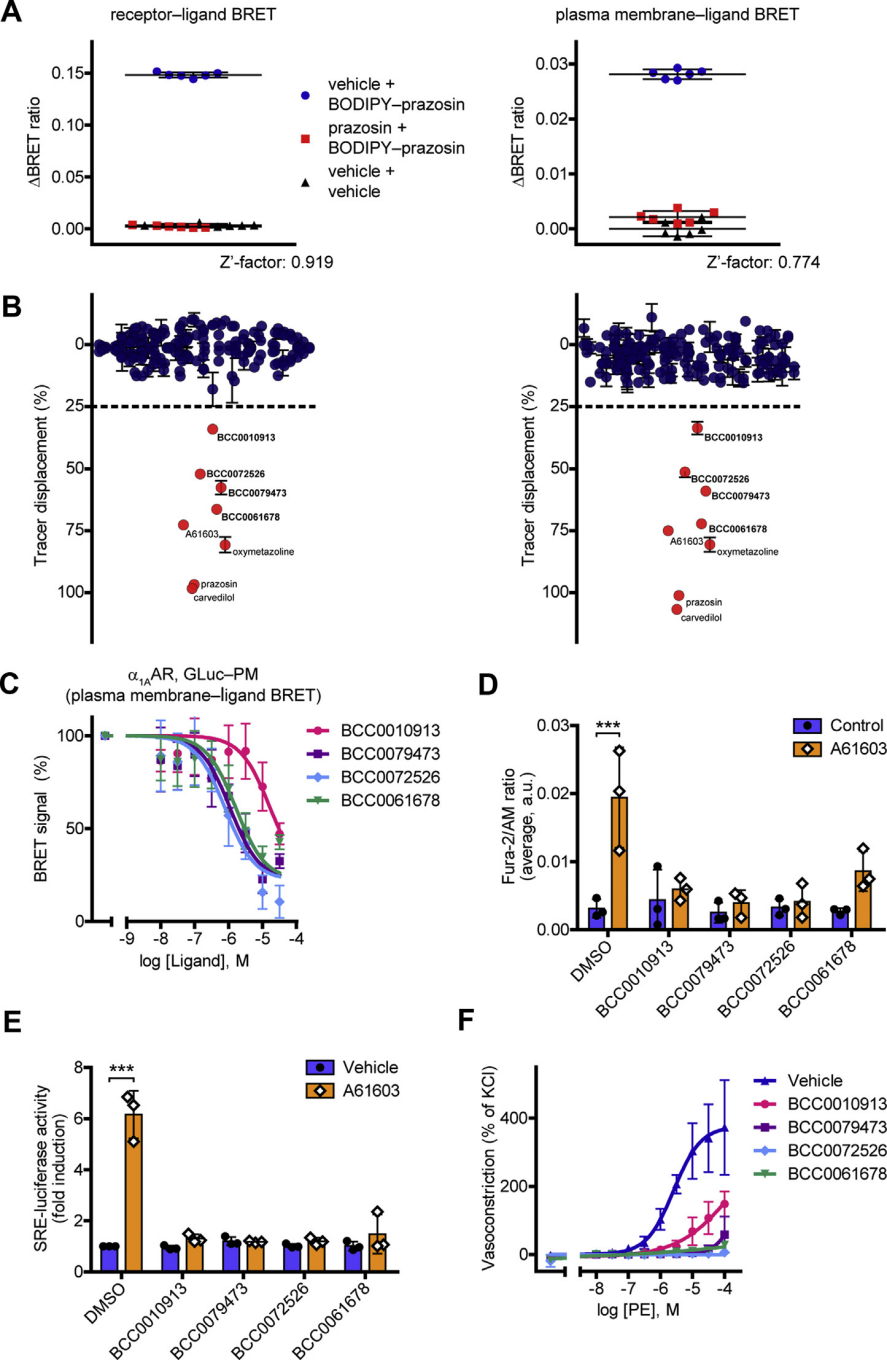
**Receptor ligand screening with GLuc BRET.***A*, statistical validation of the suitability of receptor–ligand BRET and PM–ligand BRET for high-throughput screenings. After 20-min prazosin (10 μM) or dimethyl sulfoxide pretreatment, 150 nM BODIPY FL–prazosin was added to the cells. BRET was normalized to vehicle-treatment. Z’-factor values were 0.919 (GLuc–ɑ_1A_AR) and 0.774 (ɑ_1A_AR with GLuc–PM), respectively. Results of a representative experiment with six technical replicates are shown, *n* = 3. Data are mean ± SD. *B*, screening for ɑ_1A_AR ligands. *Left panel*, receptor–ligand BRET (GLuc–ɑ_1A_AR); *right panel*, PM–ligand BRET (ɑ_1A_AR with GLuc–PM). A 180-compound library was tested, and A61603, oxymetazoline, prazosin, and carvedilol were used as positive controls. After 20-min unlabeled ligand (10 μM) pretreatment, the displacement of 150 nM BODIPY FL–prazosin was assessed. The screening was performed in duplicate, data are mean ± range. *C*, competitive ligand binding of the hits using the PM–ligand BRET. Increasing concentrations of hits and 375 nM BODIPY FL–prazosin were added to the cells, *n* = 4. Data are mean ± SD. One site competitive ligand binding curves were fitted, the K_i_ values were 1.845 μM (BCC0010913), 121.6 nM (BCC0079473), 86.12 nM (BCC0072526), and 180.4 nM (BCC0061678). *D*, effects of the hits on calcium signaling of ɑ_1A_AR. Cytosolic calcium measurements were performed in Fura-2/AM-loaded cells that overexpressed ɑ_1A_AR. The cells were first treated with the hits (10 μM) or vehicle for 1 min, then 100 pM A61603 was used as a stimulus for 3 min. Averages of the signals are shown, data are mean ± SD, *n* = 3. The compounds did not induce significant calcium signal but prevented the A61603 effect (two-way ANOVA with Tukey post hoc test, ∗∗∗*p* = 0.0001). *E*, functional activity of the hits assessed with the SRE-luciferase reporter. After 20-min pretreatment with vehicle or the tested compound (30 μM), 2-h costimulation with 1 nM A61603 or vehicle was applied. All the hits were antagonists, as they did not induce SRE-luciferase activity but prevented the A61603 effect, *n* = 3. Scatter dot plots with bars are shown, data are mean + SD (two-way ANOVA with Tukey post hoc test, performed on the nonnormalized data, ∗∗∗*p* = 0.0003). *F*, the identified new ɑ_1A_AR ligands antagonize the phenylephrine-induced vasoconstriction. Myography measurements were performed on murine aortic rings. Data are mean ± SD. Concentration–response curves were fitted. No significant effect of hit treatment on resting tension was found (one-way ANOVA with Tukey post hoc test), *n* = 3. α_1A_AR, α_1A_ adrenergic receptor; BRET, bioluminescence resonance energy transfer; GLuc, *Gaussia* luciferase; PM, plasma membrane; SRE, serum response element.

We tested whether our system could be applied for receptor ligand screening. As a model receptor, we chose the α_1A_AR, a major pharmacological target in the treatment of high or low blood pressure and benign prostate hyperplasia (29). In preliminary experiments, we compared the signal variance in equilibrium and kinetic binding measurements. From this viewpoint, the latter seemed to be more advantageous because of the possibility to apply baseline correction. To statistically characterize the suitability of our system for high-throughput screenings, we calculated the Z’-factor statistical parameter both for receptor‒ligand and PM‒ligand BRET (Fig. 5*A*) (30). It was 0.919 and 0.774, respectively, proving that our system could also be applied in high-throughput screenings. Thereafter, we made a test screening with a compound library of 180 compounds (Fig. 5*B* and Table S1). As positive controls, four known α_1A_AR ligands were also tested. We defined those compounds as hits that induced at least 25% decrease of the signal (induced 25% displacement of the tracer ligand). Four compounds were found as hits (Fig. S5), which were previously not known to bind to α_1A_AR. The hits were further characterized. Their K_D_ values were assessed using both ligand binding setups (Fig. 5*C* and Fig. S6*A*). Their functional effects on α_1A_AR, a receptor known to trigger phospholipase C–mediated phosphatidylinositol 4,5-bisphosphate (PIP_2_) hydrolysis and concomitant calcium signaling, were also investigated. All four hits were able to prevent the cytosolic calcium release induced by the α_1A_AR agonist A61603 (Fig. 5*D*). Similarly, the hits abolished the A61603-induced serum response element (SRE)-luciferase activation, a reporter of mitogen-activated protein kinase/extracellular signal-regulated kinase (MAPK/ERK) signaling and serum response factor activity (Fig. 5*E*). Consistently with these results, they shifted the A61603 concentration‒response curve of PIP_2_ cleavage to the right, representing their competitive α_1A_AR antagonistic property (Fig. S6*B*). Moreover, they alone had no effect on the vascular tone of mouse aortic rings but antagonized the vasoconstrictor effect of phenylephrine, a well-known α_1_ adrenergic receptor agonist (Fig. 5*F*). These results demonstrate that our GLuc-based assay could be utilized as a powerful novel tool for drug screening.

## Discussion

Over the last 2 decades, resonance energy transfer–based methods are emerging in pharmacological studies, which is particularly true for ligand binding investigations (8, 13). Time-resolved FRET or BRET (using NanoLuc) was successfully adapted to study various receptors, including GPCRs and receptor tyrosine kinases (9, 11, 12, 31, 32, 33, 34). Here we described a novel ligand binding assay that has the merits of previous resonance energy transfer–based methods and overcomes many of their limitations. Like other resonance energy transfer–based approaches, our system has remarkably low signal variance, is simple and fast to perform as it lacks laborious experimental steps, and is harmless to health. In addition, resonance energy transfer–based systems allow ligand binding kinetics to be monitored in real time with exceptional temporal resolution. The importance of knowledge of ligand binding kinetics cannot be emphasized enough, as several studies have shown that the *in vivo* action of drugs correlates much better with kinetic ligand properties, such as k_on_, k_off_, or ligand residence time, than with ligand parameters assessed in equilibrium. Moreover, our approach is cost-effective, since it applies the extremely bright *Gaussia* luciferase that utilizes the native coelenterazine as a substrate. It should be noted that inexpensive substrates are available for NanoLuc as well, for example, coelenterazine *h*, but the brightness produced is significantly lower.

Previous resonance energy transfer–based approaches suffered from the need for molecular biological modification of the protein of interest, since the generation of properly functioning fusion proteins sometimes requires tremendous effort and the potential risk of altered function cannot be completely ruled out even with cautious design and application of appropriate controls. Accordingly, several cases of tag- or even linker-induced functional changes for multiple receptors have been described, such as endoplasmic reticulum retention, altered ligand affinity, or modified effector coupling (12, 24, 25, 35, 36). These examples may be just the tip of the iceberg, as malfunctioning constructs are not likely to be published. To examine ligand binding of unlabeled receptors, we developed a bystander BRET-based approach. Although we experienced a notable drop in the signal amplitude, the signal deviation remained low; thus, the separation between signal and background remained high. We believe that this price is significantly outweighed by the benefit of being able to investigate unmodified receptors.

Since only the unmodified receptor and the cell surface–targeted bioluminescent sensor have to be expressed in a cell line, our assay can be readily applied to study any cell surface receptor when a fluorescent ligand is available. We adapted our system for various GPCRs and non-GPCRs, including AT_1_R, AT_2_R, α_1A_AR, β_2_AR, D_1_R, EGFR, and TfR. We prefer GLuc to NanoLuc as a bioluminescent donor because of the higher effect size in our system, but NanoLuc can also be used based on similar logic. In addition to these proteins, we always coexpressed dominant-negative dynamin to prevent possible interference caused by internalization. We found a positive correlation between the amount of receptor DNA transfected and the amplitude of the specific BRET signal. Therefore, it must be noted that the assay is more suitable for measuring ligand binding of highly expressed receptors, and endogenous receptors with low copy number may not reach the detection limit of the method. The system allows the determination of any ligand binding properties and is particularly useful for kinetic ligand binding measurements as the assay allows real-time detection. Theoretically, the signal of the PM‒ligand BRET setup may be influenced by the change in distance between the fluorescent ligand-bound receptor and the GLuc‒PM biosensor. It is known that subtle changes of the bystander BRET between luciferase-tagged receptors and PM–targeted fluorescent proteins can also reflect the lateral movement of the receptor between PM compartments (20, 37). However, we found no indication that this phenomenon would contribute significantly to the measured signal in the ligand association experiments with AT_1_R or α_1A_AR. In addition, this issue is unlikely in dissociation experiments, because alterations in the localization of the unlabeled receptor are unseen by our assay after dissociation of the fluorescent ligand.

In high-throughput applications, ligand binding assays offer several advantages over approaches for assessing signaling pathways. Receptors are known to activate multiple signaling pathways, which are not equally modulated by certain ligands (biased agonists) (38, 39, 40). In order to achieve the highest possible hit rate with functional assays, multiple signaling pathways must be simultaneously examined with distinct assays, and different assay conditions are required for agonists and antagonists. This means that a compound needs to be screened in several setups, significantly increasing the cost and the time of the screening. These disadvantages are absent in ligand binding measurements; a single setup is sufficient to perform a receptor ligand screen. We proved that our assay is suitable for high-throughput screenings. As a demonstration, we screened a compound library of 180 molecules for α_1A_AR ligands with our system. We found four previously unknown α_1A_AR ligands, which acted as α_1A_AR antagonists in multiple functional assays. The relatively high hit rate is consistent with the observation that a large number of drugs exert off-target effects through α_1A_AR, causing orthostatic hypotension and vertigo (41).

In summary, we have developed a novel ligand binding assay that allows the determination of ligand binding with low signal variance and high temporal resolution, providing a powerful tool for drug characterization and development.

## Experimental procedures

### Materials

TAMRA‒AngII was purchased from AnaSpec. BODIPY FL‒prazosin, biotinylated EGF conjugated to Alexa Fluor 488 streptavidin, and Alexa Fluor 488–conjugated human transferrin were bought from Thermo Scientific. BODIPY FL‒SKF83566 and BODIPY FL‒(S)-propranolol were from HelloBio. Candesartan and SCH 23390 were purchased from Tocris. [Sar^1^,Ile^4^,Ile^8^]-angiotensin II was bought from Bachem. The compound library consisting of randomly selected 180 molecules was from BioAscent. Native coelenterazine and coelenterazine *h* were from Regis Technologies. Furimazine and the Luciferase Assay System were from Promega; DeepBlueC (coelenterazine 400a) was from Perkin-Elmer. Fura-2/AM was from Molecular Probes. ICI118,552 EGF, transferrin, angiotensin II, A61603, prazosin, and otherwise not stated materials were from Sigma-Aldrich.

### Plasmid constructs

The plasmid constructs coding the rat AT_1_R, α_1A_AR, β_2_AR, EGFR, and the PM PIP_2_ BRET biosensor (L10‒Venus‒T2A‒PLCδ1-PH‒SLuc) were described (23, 42, 43). SRE-luciferase, containing the SRE promoter region fused with the coding sequence of firefly luciferase, was from Promega. The cDNA of human D_1_R and hemagglutinin‒dynamin2A‒K44A (DN-Dyn) were kind gifts from Dr Marc G. Caron and Dr Kazuhisa Nakayama, respectively. The coding sequence of untagged rat AT_2_R without the untranslated regions was polymerase chain reaction (PCR) amplified and inserted into pEYFP-N1 by replacing YFP. The C-terminal GFP-tag of TfR‒GFP (kindly provided by Dr Tamás Balla) was replaced by hemagglutinin-tag to create TfR‒hemagglutinin. To generate GLuc‒PM, the DNA sequence of *Gaussia* luciferase signal peptide followed by GLucM23 (humanized GLuc harboring the K50E, M60L, V113D, M127I, and G184D mutations (16)) fused to the transmembrane domain of platelet derived growth factor (PM) with a flexible (GGGSGGGSRSGGGSGGGSGGGS) linker was gBlock synthetized and was inserted into pcDNA3.1 vector. To create NanoLuc‒PM, the coding sequence of NanoLuc N-terminally fused with the secretion signal peptide of interleukin-6 was gBlock synthetized and the signal peptide-fused GLucM23 was replaced with that in GLuc‒PM. N-terminally GLuc- or NanoLuc-tagged AT_1_Rs harboring a short linker (GGGSGGGS) were generated by PCR amplification of the sequence of rat AT_1_R to replace PM in GLuc‒PM and NanoLuc‒PM, respectively. A similar strategy was used to generate GLuc‒α_1A_AR. To create GLuc‒PM‒Venus and NanoLuc‒PM‒Venus, GLuc‒PM and NanoLuc‒PM were PCR amplified without stop codons and were inserted into pVenus-N1 vector before the sequence of monomeric Venus.

### Cell culture and transfection

HEK 293T cells (from American Type Culture Collection: CRL-3216) were cultured in Dulbecco's modified Eagle's medium (DMEM) supplemented with 10% fetal bovine serum and 100 IU/ml penicillin/streptomycin. To visualize the cellular localization of the probes, the cells were plated on coverslips on the day before transfection, and the adherent cells were transfected with the calcium phosphate precipitation method: for each well (24-well plate), 1 μg plasmid DNA was diluted in 45 μl distilled water, to this 5 μl 2.5 M CaCl_2_ was rapidly added, then 50 μl 2× HEBS (Hepes-buffered salt solution, containing 274 mM NaCl, 15 mM glucose, 42 mM Hepes, 10 mM KCl, 1.4 mM Na_2_HPO_4_, pH 7.1) was added slowly, and the solution was added dropwise to the culture medium (1 ml) of cells. The amounts and volumes for calcium phosphate precipitation were proportionally increased or decreased for wells with bigger or smaller surfaces. For cytosolic calcium measurements, the cells were plated on IBIDI μ-Slide 8-well plates and a similar procedure was applied. Otherwise, the transfections were performed in suspension using the calcium phosphate precipitation method, and the cells were plated on white 96-well plates. Mixture volumes were multiplied by the number of wells transfected, plasmid DNAs were diluted in 9 μl distillated water, 1 μl 2.5 M CaCl_2_, and 10 μl 2× HEBS per well, as described above, and the transfection solution was added dropwise to HEK 293T cells suspended in supplemented DMEM (8 × 10^4^ cells in 200 μl of medium per well). A volume of 200 μl of the mixture was seeded on each well. For receptor‒ligand BRET measurements, the plasmid DNA of luciferase-tagged receptor and DN-Dyn were transfected in 1:4 ratio (0.05 and 0.2 μg per well). In the PM‒ligand BRET measurements, the receptor:luciferase‒PM:DN-Dyn plasmid DNA ratio was 20:1:5 (0.2, 0.01, and 0.05 μg per well), unless otherwise stated.

### Bioluminescent and fluorescent image acquisition

For live cell image acquisition, coverslips were held in Chamlide magnetic chambers in Hanks' balanced salt solution media. Images were collected with a Nikon Ti2 inverted microscope with a Hamamatsu ORCA Flash 4 V3 sCMOS camera using a 100× objective. Five micromolar coelenterazine *h* and 5 μM native coelenterazine were used as substrates for NanoLuc and GLuc, respectively. Luminescence was detected by applying 10-s exposure time with 4× averaging. Fluorescence was recorded by excitating at 488 nm laser, and emission was detected using a Quad band filter. The images were individually processed, looking up tables were adjusted in each image distinctively to achieve high contrast.

### Luciferase activity measurements

The emission spectra of GLuc‒PM and NanoLuc‒PM were detected using a Thermo Scientific Varioskan Flash multimode plate reader (spectral scanning mode using a monochromator). Since the peak total luminescence of GLuc surpassed the detection limit of the Varioskan Flash plate reader, fluorescence and luminescence intensities of GLuc‒PM‒Venus or NanoLuc‒PM‒Venus were measured with a BMG Labtech CLARIOstar microplate reader. Venus fluorescence was assessed by exciting at 497/15 nm and measuring emission at 540/20 nm. Total luminescence was measured without filter (gain 1100, integration time 0.2 s).

### Bioluminescence resonance energy transfer measurements

All measurements were performed on adherent cells. On the day after transfection, the cells were washed and the medium was changed to modified Krebs–Ringer medium (120 mM NaCl, 10 mM glucose, 10 mM Na-Hepes, 4.7 mM KCl, 1.2 mM CaCl_2_, 0.7 mM MgSO_4_, pH 7.4). Equilibrium binding studies were performed after 2-h incubation of labeled and unlabeled ligands at room temperature. Incubations were made on ice for β_2_AR and D_1_R binding measurements. Luminescence was measured using a Varioskan Flash multimode plate reader at 27 °C. For NanoBRET measurements, light intensities were measured with 460/20- and 610/60-nm bandpass filters after addition of 5 μM coelenterazine *h*. GLuc BRET measurements were performed after treatment with 5 μM native coelenterazine, TAMRA‒AngII binding was monitored using 480/20- and 610/60-nm filters, whereas 480/20- and 530/20-nm filters were applied in BRET measurements with green-emitting (BODIPY FL- or Alexa Fluor 488–conjugated) fluorescent ligands.

To assess K_off_ of fluorescent ligands, the ligands were preincubated with the cells for 15 min. Thereafter, the cells were washed and the medium was replaced with media containing coelenterazine and unlabeled ligand (10 μM candesartan for AT_1_R, 10 μM prazosin for α_1A_AR) to prevent rebinding. To determine the maximal signal, control cells were treated with labeled ligand and coelenterazine, but no unlabeled ligand was added. Basal BRET was assessed in cells treated with no labeled ligand. For analysis, basal BRET was always subtracted. For K_on_ measurements of fluorescent ligands, cells were treated with coelenterazine, baseline BRET was monitored for 3 min, and then the cells were treated with fluorescent ligands. Kinetic parameters of unlabeled ligands were measured similarly. After baseline BRET assessment, the cells were treated simultaneously with fluorescent ligand and increasing concentrations of unlabeled ligand.

PM PIP_2_ levels were monitored using a PIP_2_ BRET biosensor (L10–Venus–T2A–PLCδ1-PH–SLuc) (23). Bystander BRET was measured between Super *Renilla* luciferase-tagged PLCδ1-PH (the PIP_2_-binding domain of PLCδ1) and PM–targeted Venus in cells coexpressing PIP_2_ BRET biosensor and α_1A_AR. The BRET ratio decreased upon treatment with α_1A_AR agonist, which reflects the phospholipase C–mediated PIP_2_ cleavage. A 5-min pretreatment with the hit compounds (10 μM) was applied, then the cells were stimulated with increasing concentrations of α_1A_AR agonist A61603. First, the agonist-induced BRET change was determined (the BRET ratio after pretreatment was subtracted from the average of the BRET ratios measured in the first 5 min after stimulation), then the BRET signal was expressed in the percent of the 10 μM A61603-induced BRET change in vehicle-pretreated cells.

The screening was performed using a compound library harboring 180 compounds (Table S1). The compounds were randomly recruited from the BioAscent library of approximately 1.2 × 10^6^ molecules. To obtain a better signal-to-noise ratio, 1.75 times more cells (1.4 × 10^5^) were seeded per well on 96-well plates. Outer wells of plates were not used to rule out the edge effect; only the inner 60 wells were measured. Before the measurements, the cells were pretreated with the compounds for 20 min in 10 μM (which was diluted to 6 μM after substrate and ligand treatment). After addition of coelenterazine, baseline BRET was determined for 5 min, then the cells were stimulated with 150 nM BODIPY FL–prazosin or vehicle. The same experimental procedure was used for Z’-factor assessment, BRET was monitored in six wells per condition on 96-well plates, and the Z’-factor was calculated as previously described (30).

### Cytosolic calcium concentration measurements

Adherent HEK 293T cells overexpressing α_1A_AR were treated with 200 μM sulfinpyrazone and loaded with the ratiometric fluorescent dye Fura-2/AM (2 μM, 45 min). Single-cell calcium measurements were performed using an inverted microscope (Axio Observer, Zeiss) equipped with a 40× oil immersion objective (Fluar, Zeiss) and a Cascade II camera (Photometrics). Excitation wavelengths were set by a random access monochromator connected to a xenon arc lamp (DeltaRAM, Photon Technology International). For ratiometric measurements of Fura-2 excitation wavelengths of 340 and 380 nm were selected combined with a 505-nm dichroic filter and a 525/36-nm emission filter set. Data acquisition was performed by the MetaFluor (Molecular Devices) software. Images were acquired every 5 s. After a control period (1 min), cells were treated with vehicle (dimethyl sulfoxide) or test compound (10 μM), then the cells were stimulated with 100 pM A61603 for 3 min. Image analysis was performed with ImageJ software; the ratio of fluorescent intensities measured at 340 and 380 nm excitations was taken. About 150 to 200 regions of interest representing individual cells from each experiment and each treatment were measured, and fluorometric ratios were normalized to the control period.

### SRE-luciferase activity measurement

HEK 293T cells were transiently transfected with SRE-luciferase and receptor plasmids in suspension and were seeded on 96-well white plates. On the next day, the cells were serum starved for 2 h by replacing the media to DMEM containing 1% penicillin/streptomycin and 1% bovine serum albumin. Then the medium was changed to modified Krebs–Ringer medium, and the cells were pretreated with vehicle or the investigated compounds (30 μM) for 20 min, then 2-h costimulation with 1 nM A61603 or vehicle was applied at 37 °C. Then the cells were plated on ice and were lysed with passive lysis buffer (Promega). After 20-min rocking at room temperature, the Luciferase Assay System (Promega) substrate was added and luminescence was measured with a Thermo Scientific Varioskan Flash multimode plate reader at 27 °C.

### Wire myography

Myography experiments were performed as described (44). Male C57BL/6 mice were terminated by cervical dislocation, and the vascular system was perfused through the left ventricle and a right atrial slit with Kreb’s solution containing 119 mM NaCl, 4.7 mM KCl, 2.5 mM CaCl_2_, 1.17 mM MgSO_4_, 20 mM NaHCO_3_, 1.18 mM KH_2_PO_4_, 0.027 mM EDTA, and 10 mM glucose (pH 7.4). Abdominal aortas were removed and placed into Kreb’s solution. Aortic rings (3–4 mm), five segments from each animal, were mounted onto a multichamber isometric myograph system (Danish Myo Technology). The organ chambers were filled with Kreb’s solution kept at 37 °C and bubbled with carbogen gas (5% CO_2_ and 95% O_2_). Recording of isometric tension was performed with the Powerlab data acquisition system and the LabChart evaluation program (ADInstruments). The segments were allowed to equilibrate for 30 to 40 min, and the resting tension was adjusted to 10 mN. First, contractions were elicited by hyperkalemic (124 mM) solution for 1 min. Endothelial integrity was tested with the vasodilatory response by acetylcholine (100 nM) after phenylephrine precontraction (100 nM). Before compound treatments, a 3-min KCl reference contraction was induced, then resting tension was adjusted again with washing steps. Treatments with the hit compounds (10 μM) were applied for 15 min. Next, phenylephrine was administered in cumulative concentrations (10 nM–100 μM). Vasoconstrictor responses were expressed as percentages of the 3-min KCl-induced reference contraction. All experimental procedures were reviewed and approved by the Animal Care Committee of Semmelweis University (Budapest, Hungary).

### Mathematical and statistical analysis

Data plotting, statistical analysis, and curve fittings were performed with GraphPad Prism 9 software. One or two site-specific binding curves were fitted on the specific signal points of equilibrium binding measurements. With the use of the determined K_D_ values, total (one site or two site) and nonspecific binding were also fitted. One or two site competition binding curves were fitted in the case of equilibrium competitive ligand binding measurements. K_i_ values were calculated with the Cheng–Prusoff equation (45). To determine labeled ligand–receptor K_off_ in measurements of Figure 4, one phase exponential decay curves were fitted. Thereafter, the “association kinetic (one ligand concentration)” equation was used to calculate labeled ligand–receptor K_on_ with constrained K_off_ and B_max_ (maximum specific binding, determined in equilibrium binding measurements). K_off_ and K_on_ values of the unlabeled ligand–receptor interaction were determined with the “Kinetics of competitive binding” (Motulsky–Mahan) equation (28). To calculate τ, one phase association curves were fitted. The results of curve fits for ligand binding experiments in the main figures are summarized in Table S2.

For statistical comparison, unpaired two-tailed *t* test and one-way or two-way ANOVA with Tukey post hoc test were applied.

## Data availability

GLuc‒PM and NanoLuc‒PM biosensors will be available from Addgene after publication.

## Conflict of interest

The authors declare that they have no conflicts of interest with the contents of this article..
