# Supplementary material for: A general method for quantifying ligand binding to unmodified receptors using Gaussia luciferase
Source: J Biol Chem. 2021 Feb 2;296:100366. doi: 10.1016/j.jbc.2021.100366 (PMC7950324; doi:10.1016/j.jbc.2021.100366)
Supplement: Supplemental Figures S1–S6 and Tables S1–S2 [file mmc1.pdf]

Sensitive detection of ligand binding parameters of unmodified receptors using *Gaussia* luciferase

András Dávid Tóth, Dániel Garger, Susanne Prokop, Eszter Soltész-Katona, Péter Várnai,
András Balla, Gábor Turu, László Hunyady

Supporting Information:

Figure S1…………………………………………………………………..………………………S-1
Figure S2……………………………………………………………………………..………………S-2
Figure S3……………………………………………………………………………………..………S-3
Figure S4………………………………………………………………………………………..…S-4
Figure S5…………………………………………………………………..………………………S-5
Figure S6……………………………………………………………………………………………..S-6
Table S1……………………………………………………………………………………..……………S-7
Table S2…………………………………………………………………………………………….S-11

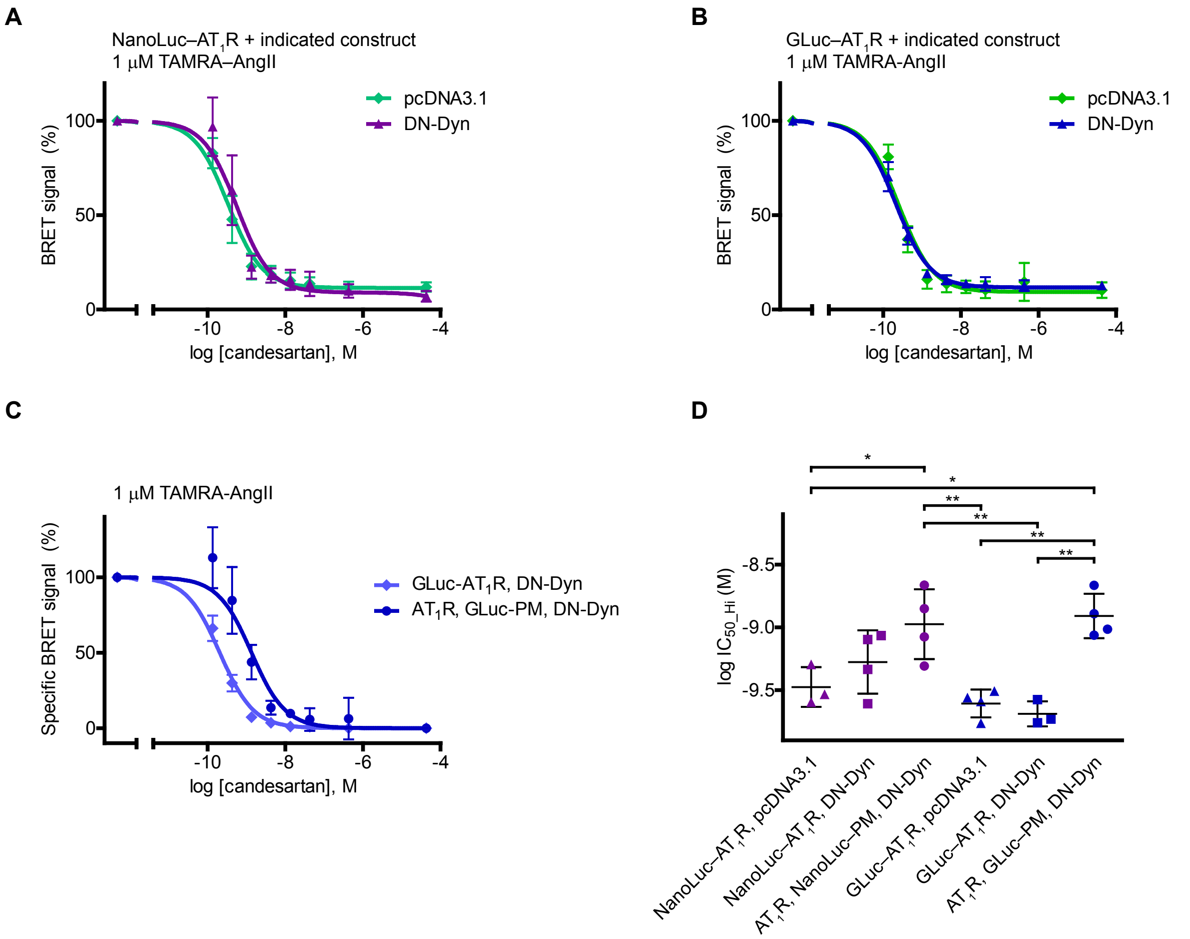

Figure S1. Comparison of binding curves obtained with different BRET ligand binding setups. Equilibrium binding measurements were performed, 1 µM TAMRA–AngII and increasing concentrations of candesartan were used. Two site competitive ligand binding curves were fitted. *A* and *B*, Ligand binding of NanoLuc–AT_1R (A) and GLuc–AT_1R (right) was measured in the presence or the absence of HA-dynamin2A-K44A (DN-Dyn) co-expression. *C,* Comparison of the specific signal curves measured with receptor–ligand BRET or plasma membrane–ligand BRET. Cells were transfected with the indicated constructs. Non-specific signals were subtracted from the total signal. *D,* N-terminally tagged AT_1Rs have altered ligand binding properties. Log IC_{50_Hi} values were assessed in each independent experiment measured with different setups. One-way ANOVA, Tukey post-hoc test. *P* values are (from top to bottom and left to right): *,0.0417; *,0.0179; **, 0.0039; **, 0.0026; **, 0.0015; **, 0.0011, n = 3-4. Data are mean ± SD.

Figure S2

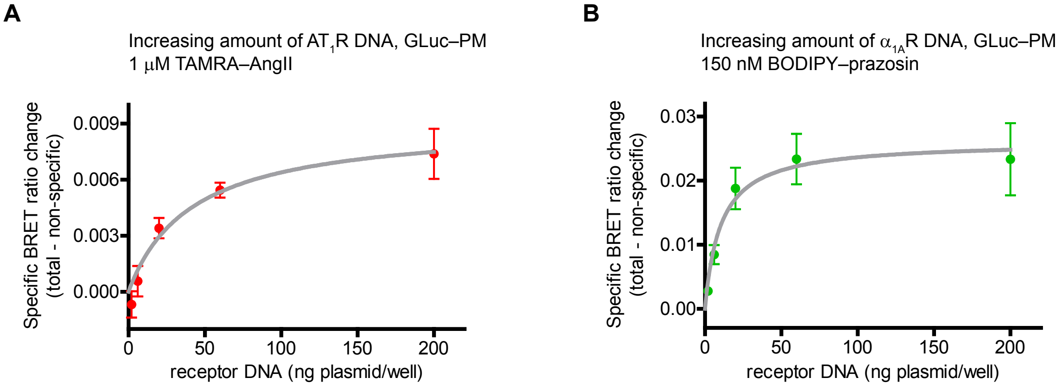

Figure S2. Correlation between the amplitude of the specific signal and the amount of receptor DNA transfected in plasma membrane–ligand BRET measurements. Cells were co-transfected with GLuc–PM, DN-Dyn, and increasing amounts (2, 6, 20, 60, and 200 ng) of the indicated plasmid. The total amount of DNA was kept constant by addition of pcDNA3.1 empty vector. Cells were treated with the appropriate fluorescent ligand with or without unlabeled ligand (10 μM candesartan for AT_1R or 10 μM prazosin for $\alpha_{1A}AR$) to determine the total and the non-specific signal, respectively. The specific BRET ratio was calculated by subtracting the non-specific signal from the total signal. Data are mean ± SD, *n* = 3. One site specific binding curves were fitted, R^2 values were 0.9134 (A) and 0.8721 (B).

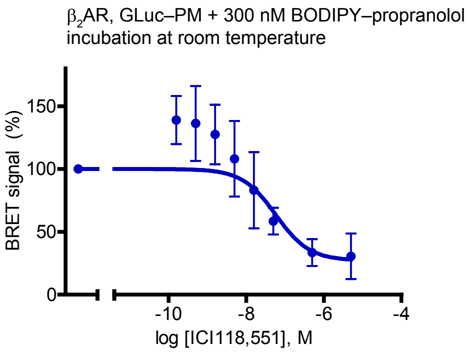

Figure S3. Ligand binding of β_2AR measured with plasma membrane–ligand BRET. Incubation was made at room temperature. Data are mean ± SD, presented as the percentage of the ΔBRET ratio induced by the treatment with BODIPY FL–(S)-propranolol without unlabeled ligand (0.0146 ± 0.0026 was 100%). One site competitive ligand binding curve was fitted, $n = 4$. $R^2 = 0.5852$.

Figure S4

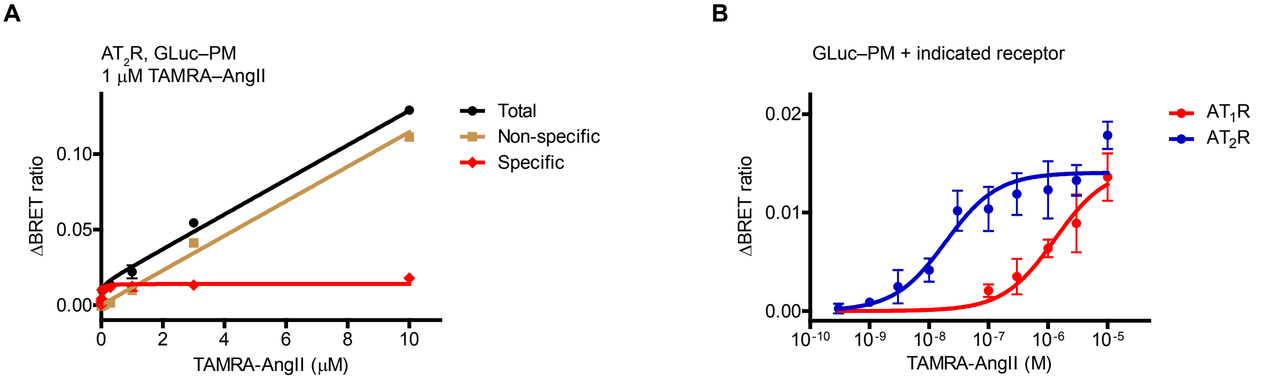

Figure S4. Affinity of TAMRA–AngII for AT_2R assessed with plasma membrane–ligand BRET. Cells co-expressing AT_2R, GLuc–PM, and DN-Dyn were incubated with increasing concentrations of TAMRA–AngII. Non-specific signal was determined by the addition of an excessive amount of non-labeled competitor ligand (100 μM AngII), specific signal was calculated by subtracting the non-specific signal from the total signal. *A*, Since application of two site binding equations resulted in ambiguous fits, one site specific, one site total and non-specific binding curves were fitted. K_D of specific binding was 19 nM. *B*, Relation of specific TAMRA–AngII binding to AT_1R and AT_2R. To simplify the comparison, we fitted one-site specific binding curve also for AT_1R (data of Fig. 2L was used). The K_D of TAMRA–AngII binding to AT_1R was 1.295 μM. Data are mean ± SD, $n = 3$.

Figure S5

BCC0010913
(E)-3-(1,3-Benzoxazol-2-yl)-1-[4-(1H-indol-3-yl)piperidin-1-yl]prop-2-en-1-one
PubChem CID: 41710697

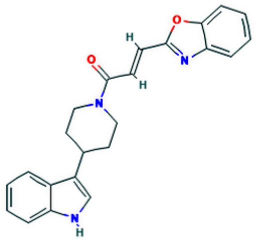

BCC0079473
1-(2,3-Dihydro-1,4-benzodioxin-6-yl)-3-[2-(4-phenylpiperazin-1-yl)ethyl]urea
PubChem CID: 22430427

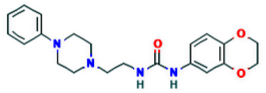

BCC0061678
1-(3,4-Dihydro-1H-isoquinolin-2-yl)-2-[4-(5-fluorobenzotriazol-1-yl)piperidin-1-yl]ethanone
PubChem CID: 644644

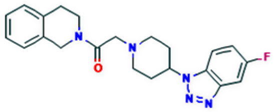

BCC0072526
N-(3,5-Dimethylphenyl)-2-[[5-(4-methoxyphenyl)-1H-1,2,4-triazol-3-yl]sulfanyl]acetamide
PubChem CID: 1075955

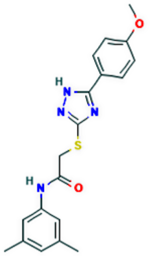

Figure S5. Name and chemical structure of the hits of the α_{1A}AR ligand screen. The images are from PubChem.

Figure S6

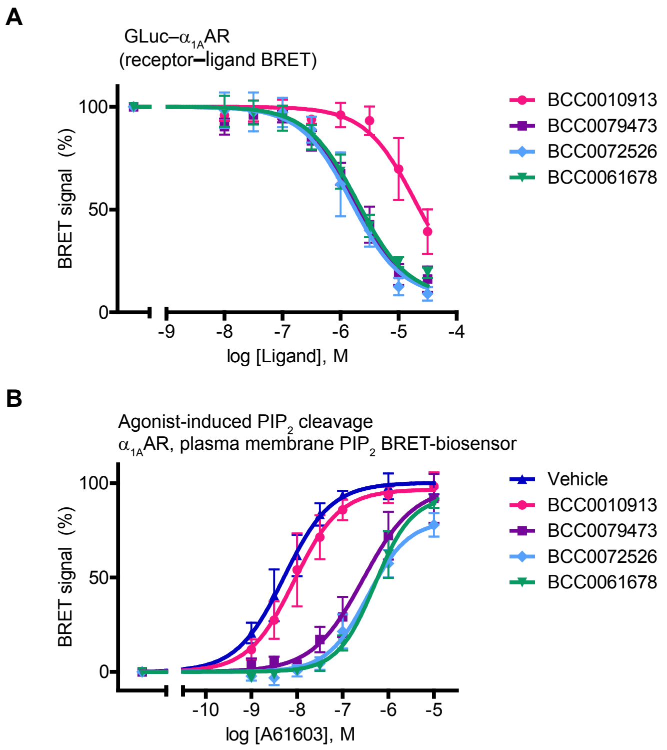

Figure S6. *A,* Competitive ligand binding of the hits to GLuc-tagged α_{1A}AR assessed with the receptor–ligand BRET setup. Increasing concentrations of hits and 375 nM BODIPY FL–prazosin were added to the cells, $n = 4$. Data are mean ± SD. One site competitive ligand binding curves were fitted, the K_i values were 2.747 µM (BCC0010913), 248.2 nM (BCC0079473), 214.9 nM (BCC0072526), 274.3 nM (BCC0061678). *B,* Antagonistic effects of the identified hits on the concentration–response curve of A61603 in PIP_2 hydrolysis measured with BRET, $n = 4$. Data are mean ± SD.

Table S1. Results of the α_{1A}AR ligand screening with receptor–ligand and plasma membrane–ligand BRET set-ups.

Compound Id	SMILES	receptor-ligand BRET decrease (%) Replicate1	receptor-ligand BRET decrease (%) Replicate2	plasma membrane-ligand BRET decrease (%) Replicate1	plasma membrane-ligand BRET decrease (%) Replicate2
BCC0070184	O=C(NC1CCCC1)CCCCCN1C(=O)c2c(C1=O)cccc2	-0,27	-0,57	-3,55	-2,96
BCC0107331	O=C(Nc1sc(c(n1)c1ccc2c(c1)CCN2C(=O)c1ccc(cc1)F)C)CN1CCCC1	0,64	-1,56	-6,47	-8,25
BCC0095885	COc1ccc(c(c1)C1NC(c2cc(OC)ccc2OC)C(C(C1)(O)c1ccccc1OC)C)OC	-7,52	-7,49	-4,41	-7,15
BCC0039164	O=C(Cn1nnnc1C)Nc1ccc(cc1)[N+](=O)[O-]	-5,72	-3,90	-1,39	-4,72
BCC0076237	COc1cc(CCNC(=O)C(=O)Nc2ccc(c(c2)Cl)Cl)ccc1OC	-4,58	-6,30	-3,25	0,03
BCC0050675	CCOC(=O)CSc1cc(C)nc(n1)NC(=O)C	-5,24	-5,52	-1,77	-4,26
BCC0067850	O=C(c1ccc(cc1)Oc1ccccc1)NCCOCc1ccccc1	-4,17	-4,98	-0,88	-3,66
BCC0095342	O=C(NC1CCCCC1)CSc1nc2c([nH]1)nccc2	-1,98	0,18	4,17	-0,65
BCC0085471	Cc1ccc(cc1)S(=O)(=O)NN1C(=O)c2ccccc2NC1c1cc(Cl)cc(c1O)Cl	0,61	-1,47	-9,99	-9,75
BCC0106461	O=C(c1ccccc1)CSc1nnc(o1)c1c[nH]c2c1cccc2	0,89	-2,96	-5,96	-3,84
BCC0045024	N#CC1=C(CCC(C1)(C#N)c1ccccc1)NC(=O)c1ccccc1	-0,31	-1,75	1,78	-3,26
BCC0069167	Cc1ccc(c(c1)C)OCCCCNC1CCCCC1	-3,81	-1,42	-10,80	-12,01
BCC0049452	OCCNC(=O)CSc1nc2ccccc2c(=O)n1C	-2,49	-0,27	-16,52	0,55
BCC0002314	COc1ccc2c(c1)[nH]c(n2)SCc1csc(n1)NC(=O)C	-0,84	-3,14	4,65	1,41
BCC0055145	COc1ccc(cc1)S(=O)(=O)CCC(=O)Nc1ccc(cn1)C	-5,34	-1,49	0,69	1,08
BCC0054499	COc1ccc(cc1C1=NOC(C1)C(=O)N1CCN(CC1)c1ccc(cc1)F)OC	-11,16	-24,81	-14,46	-9,03
BCC0093180	CN(C(=O)c1cn(nc1c1cccnc1)c1ccccc1)C	1,21	-10,67	2,88	4,63
BCC0074008	O=C(Nc1ccc(cc1)OCc1ccccc1)OCCn1ncc2c(c1=O)cccc2	-23,44	-1,91	3,16	3,95
BCC0076402	Clc1ccc2c(c1)nc(o2)c1ccccc1Cl	3,60	-9,30	3,30	4,36
BCC0020765	Clc1cnc2c(c1)[nH]c(n2)SC(c1nc2ccccc2c(=O)[nH]1)C	-4,48	-0,92	4,23	-2,51
BCC0071814	COc1cccc(c1)NC(=O)N1CCN(CC1)c1ncccc1C(F)(F)F	2,81	-5,46	0,38	-5,22
BCC0062916	Cc1ccc(c(c1)Br)OCCOCCN1CCOCC1	-8,33	-6,65	-7,36	-8,94
BCC0068009	COc1cc(ccc1OC)C(=O)Nc1ccc(cc1)C(=O)OC1CCCCC1	-12,73	-7,78	-11,50	-17,16
BCC0057387	O=C(OC(C)(C)C)NC(C(=O)Nc1ccc(cc1)Br)Cc1c[nH]c2c1cccc2	-0,14	-4,02	-1,14	-0,91
BCC0101075	O=C(N1CCC(CC1)n1c(=O)oc2c1cccc2)Cn1nnc2c1cccc2	-11,52	-13,59	-4,37	-10,76
BCC0036095	O=C(Nc1cccc(c1)[N+](=O)[O-])CN1CCN(CC1)CC(=O)Nc1cccc(c1)[N+](=O)[O-]	-5,43	-9,52	-1,40	3,14
BCC0066678	Clc1cccc(c1)N1CCN(CC1)C(=O)c1noc2c1COc1c2cccc1	-6,37	-6,61	-4,28	-2,43
BCC0094047	COC(=O)Nc1nn(c(c1)C)c1ccccc1	0,14	-3,40	1,19	4,48
BCC0072253	COc1ccc(cc1)CNC(=O)C1CN(C(=O)C1)c1ccc(cc1)OCC(=O)N1CCCC1	1,86	3,97	0,52	0,27
BCC0052396	CCOC(=O)CC(c1cccc(c1)[N+](=O)[O-])NC(=O)c1ccc(cc1)C(C)(C)C	-5,58	-1,61	3,76	3,35
BCC0111955	CCOC(=O)c1ccc(cc1)NC(=O)Nc1cnn(c1)Cc1ccc(cc1)Br	-4,40	-8,81	-1,21	-1,18
BCC0085903	Clc1ccc2c(c1)c1nnsc1CS2(=O)=O	-10,72	-5,11	-16,02	-14,64
BCC0075998	Cc1cc(C)n2c(n1)c(Br)c(n2)c1ccccc1	-14,28	-10,86	-3,26	-1,64
BCC0093092	COc1cc(SC)ccc1C(=O)Nc1nnc(s1)C(F)(F)F	0,57	-10,45	-6,52	-12,85
BCC0034087	O=C(OC(C)(C)C)NCCNC(=O)C1CCN(CC1)c1nccc(n1)C(F)(F)F	-1,31	-1,75	-0,85	5,19
BCC0093696	O=NC1N(c2ccccc2)C(=O)N(C1=O)c1ccccc1	-8,11	-3,50	1,89	6,88
BCC0030379	O=C1NC(Nc2c1cccc2)c1ccc(s1)[N+](=O)[O-]	-4,92	-2,45	5,92	5,82
BCC0083297	CCC1Sc2cc(ccc2NC1=O)S(=O)(=O)CCC(=O)Nc1ccc(cc1C)Cl	-8,53	-7,56	4,69	6,72
BCC0008909	CC(c1ccc(cc1)n1ncnc1)NC(=O)C1CC1C	-5,80	-1,11	4,15	-0,30
BCC0061317	Clc1cc(Cl)c2c(c1)c(=O)n(c(n2)CN1CCOCC1)c1ccccc1C(F)(F)F	-5,91	-7,79	1,66	5,89
BCC0058402	CCCN1CCC(CC1)N1CCN(CC1)S(=O)(=O)c1ccc(cc1)C	-6,67	-6,34	-0,92	2,58
BCC0102215	COc1ccc(cc1)CNc1onc(n1)c1ccccc1F	-11,96	-8,15	-4,51	-1,93
BCC0018276	CC(=O)/C=C/1\N(C)c2c(C1(C)C)cc(c(c2)[N+](=O)[O-])N	-9,77	-8,44	-2,79	-3,46
BCC0006744	N#CC1=C(N)OC2=C(C1c1ccc(cc1)F)CN(C/C/2=C\c1ccc(cc1)F)C(=O)OC(C)(C)C	-8,13	-12,94	-5,12	-7,13
BCC0063211	CN1CCN(CC1)C(=O)c1ccc(cc1)n1nc(c2c1CCCC2)C(F)(F)F	-6,27	0,26	3,59	4,59
BCC0048283	COc1cc(ccc1OC)C(=O)NCCN1CCN(CC1)C(=O)c1ccc(c(c1)OC)OC	-5,39	-5,11	0,72	3,82
BCC0089082	FC(c1cccc(c1)OCCn1cncn1)(F)F	-10,15	-3,52	2,21	4,76
BCC0037177	COc1cc(/C=C/2\N(C)C(=S)N(C2=O)c2ccccc2)cc(c1OC)OC	-12,47	-2,26	4,01	7,07
BCC0074399	OCCNC(=C1C(=O)CC(CC1=O)(C)C)Cc1ccc(c(c1)OCC)OCC	-6,98	-5,74	3,29	1,80
BCC0046853	Cc1cc(C(F)F)n2c(n1)c(cn2)C(=O)NCc1ccccc1Cl	-6,22	-13,25	0,34	-3,44
BCC0053797	COc1cc(ccc1OC)NC(=O)C1CCN(CC1)S(=O)(=O)c1cccc2c1nsn2	-11,02	-10,37	0,91	-2,85
BCC0040747	Fc1ccc(cc1)C(N(C(=O)CN1C(=O)c2c(S1(=O)=O)cccc2)Cc1cccs1)C(=O)NC1CCCCC1	-14,02	-11,31	-5,58	-7,96
BCC0045533	Fc1cc(CNC(=O)c2cc(nn2C)C(C)(C)C)c2c(c1)COCO2	-11,87	-10,57	-3,92	-6,82
BCC0023077	OCCN(Cc1ccccc1)CC(CO)O	-14,94	-14,33	-7,04	-10,41
BCC0020603	Cc1cc(C)c2c(c1)C(O)(C(=O)N2)C(F)(F)F	-0,39	3,10	0,63	0,63
BCC0068059	CCCC(=O)N1CCN(CC1)c1ccc(cc1)F	3,45	6,94	0,96	-3,32

Compound Id	SMILES	receptor-ligand BRET decrease (%) Replicate1	receptor-ligand BRET decrease (%) Replicate2	plasma membrane-ligand BRET decrease (%) Replicate1	plasma membrane-ligand BRET decrease (%) Replicate2
BCC0029746	COC(=O)C(NS(=O)(=O)c1cc(cc(c1)C(F)(F)F)C(F)(F)F)CCSC	3,64	-0,16	-1,25	-4,34
BCC0004890	COc1ccc(cc1NS(=O)(=O)c1c(C)noc1C)Cl	2,65	-2,12	-2,09	-6,17
BCC0061230	COc1ccc(cc1)N1C(=O)CC(C1=O)n1c(CO)nc2c1cccc2	-1,26	0,94	-8,89	-8,69
BCC0069026	CCOc1c(O)ccc(c1[N+](=O)[O-])C1NC(=O)N(C(=C1C(=O)OC)C)C	1,08	-2,14	-12,56	-14,15
BCC0044172	COc1cc(ccc1OC)CCn1c(SCC(=O)c2ccccc2)nc2c(c1=O)scc2	0,00	-3,07	-13,07	-16,99
BCC0078202	COc1ccc(cc1)CC(C(=O)NCc1ccccc1)CC(=O)O	2,21	1,32	-3,38	-0,19
BCC0051836	CCCCCCC1C(=C(N)OC2=C1C(=O)CCC2)C#N	2,44	2,58	-0,70	0,68
BCC0080266	CN1CCN(CC1)c1ncnc2c1oc1c2cc(cc1)Cl	2,97	5,07	-1,04	-1,87
BCC0039327	O=C(Nc1cc(cc2c1cc(o2)c1ccccc1)[N+](=O)[O-])Cn1cnnn1	4,23	5,32	-2,62	-3,69
BCC0076253	CN(CCCNCc1ccc2c(c1)OCO2)C	0,86	6,02	-1,24	-0,43
BCC0113627	CN1CCN(CC1)C(=O)COc1ccc(cc1C)S(=O)(=O)NCc1ccccc1	-2,26	0,41	1,05	-3,63
BCC0092904	O=C(N1CCC2(CC1)OCCO2)CN1CCC(CC1)N1CCCCC1	-1,80	0,52	-4,89	-1,52
BCC0083047	COc1ccccc1NS(=O)(=O)c1ccc2c(c1)SC(C(=O)N2)C	-4,91	-5,60	-3,44	-2,12
**BCC0061678**	Fc1ccc2c(c1)nnn2C1CCN(CC1)CC(=O)N1CCc2c(C1)cccc2	-65,57	-67,22	-72,24	-72,20
BCC0038731	CC(=O)Nc1cc(Cl)c(c(c1)Cl)c1ccc(o1)[N+](=O)[O-]	-7,71	-9,38	-9,56	-14,81
BCC0034820	C#CCOc1c(Cl)cc(cc1Cl)/C=C/1\S/C(=N/c2ccc(cc2)C(=O)O)/N(C1=O)C	-6,28	-5,91	-9,96	-10,16
BCC0060418	Cc1cc(cc(c1C)S(=O)(=O)N(C)C)C(C)(C)C	-0,94	-0,43	-0,99	0,23
BCC0048830	O=C(Nc1cccc(c1)C(F)(F)F)CSc1nnc(n1C)C(NC(=O)c1ccc(cc1)Cl)C	-1,30	-1,76	-2,00	-0,05
BCC0073062	O=C(c1ccccc1F)NC1CCN(CC1)S(=O)(=O)c1ccc(cc1)C(=O)O	-0,01	0,59	-2,28	-2,70
BCC0108463	O=C(Nc1nnc(s1)C)CSc1nnc(n1CCc1ccccc1)c1ccoc1C	2,82	2,31	-4,64	-0,47
BCC0102701	O=C(N1c2ccccc2Sc2c1cccc2)NCc1ccccn1	-3,47	-0,90	3,71	-0,22
BCC0100545	OC1CCN(CC1)c1ncc(c(n1)c1ccco1)c1onc(c1C)C	0,49	-1,21	-5,70	-6,85
BCC0089465	CNS(=O)(=O)c1ccc(cc1)NC(=O)Nc1cccc(c1)F	-4,81	-2,43	-7,38	-2,19
BCC0083859	Nc1cc(nc2n1nc(c2)c1ccccc1)c1ccccc1	-2,44	-5,65	-8,97	-3,51
BCC0035218	COc1cccc(c1)NC(=O)C1CCCCC1C(=O)O	-1,11	-6,10	-5,26	-9,53
BCC0055456	CN(Cc1ccccc1Sc1cccc2c1OCO2)C	3,04	3,19	-2,84	-0,37
BCC0030709	N#CCCN(S(=O)(=O)c1cccc2c1nsn2)Cc1cccs1	5,04	2,61	2,93	5,65
BCC0097050	Clc1ccc(cc1)CS(=O)(=O)N1CCN(CC1)C(=O)c1ccco1	1,56	1,59	9,49	9,32
BCC0077404	COC(=O)c1ccccc1NS(=O)(=O)c1ccc(c(c1)Cl)OC	2,35	-1,38	-4,59	-4,94
BCC0110266	Cc1ccc(cc1)CN(S(=O)(=O)c1ccc(cc1)Cl)CC(=O)N1CCOCC1	-0,21	-0,23	-5,22	-5,76
BCC0092485	O=C(Nc1ccccc1n1cccc1)Cn1nnc(n1)c1ccc(cc1)S(=O)(=O)N1CCCC1	1,13	6,72	-4,33	-11,85
BCC0106500	O=C(C1CCCN1S(=O)(=O)c1ccc2c(c1)cccc2)NCc1ccco1	-1,13	-4,84	-5,44	-16,24
BCC0065826	Fc1ccc(cc1)C(=O)NC(C(=O)O)Cc1c[nH]c2c1cccc2	4,21	3,66	5,56	6,58
BCC0074150	CCC(N1C(=O)C2C(C1=O)CCCC2)C(=O)O	3,13	5,95	3,71	-3,95
BCC0034725	N#CC1=C(N)Oc2c(C1c1scc(c1)Br)ccc(c2)N(C)C	3,11	3,69	1,07	-1,34
BCC0100557	Cc1noc(c1)c1cnc(nc1C(C)C)NCCN1CCOCC1	5,65	4,17	1,33	0,73
BCC0057676	COc1cc(CN2CCN(CC2)C(=O)c2ccccc2)cc(c1O)OC	7,63	5,18	-1,51	-1,61
BCC0010576	COc1ccc(cc1F)CN(CCS(=O)(=O)c1ccc(cc1)Cl)C	3,23	3,34	8,57	-0,14
BCC0037630	CN(c1ccc(cc1)/C=N/n1c(O)nnc1O)C	0,96	-2,99	-0,89	-6,22
BCC0020823	Cn1ccnc1SCc1nc(N)c2c(n1)cccc2	5,34	3,26	-7,86	-8,38
BCC0056899	COc1ccc(cc1)NC(=O)c1ccc(cc1)CN1CCOCC1	-2,24	3,48	-10,07	-10,30
BCC0121856	Brc1ccc2c(c1)c1nc3ccccc3nc1n2CCN1CCOCC1	-0,48	1,02	-8,33	-14,91
BCC0023706	O=C(Nc1sc2c(c1C(=O)N)CCCC2)CSc1nnc(n1C)Cc1cccs1	-1,24	6,02	1,14	-1,18
BCC0117056	CC(Nc1nnc(s1)Sc1ncnc2c1ccs2)(C)C	6,52	6,57	-11,61	-0,30
BCC0080607	CSc1ccc(cc1)CNC1CN2CCC1CC2	8,06	8,14	-0,99	2,80
BCC0059938	CCC(=O)c1ccc(cc1)OCCN1CCOCC1	8,85	5,99	-1,24	-1,63
BCC0067280	COc1cc(CCNC(=O)c2cccc3c2cccc3)ccc1OC	9,98	-0,50	0,24	-2,94
**BCC0010913**	O=C(N1CCC(CC1)c1c[nH]c2c1cccc2)/C=C/c1nc2c(o1)cccc2	-33,31	-34,88	-31,01	-36,12
**BCC0072526**	COc1ccc(cc1)c1nnc([nH]1)SCC(=O)Nc1cc(C)cc(c1)C	-51,95	-52,42	-49,30	-53,50
BCC0018636	COC(=O)c1ccc(s1)C(=O)Nc1c(C)nn(c1C)c1ccccc1	-0,35	7,75	-2,58	-0,80
BCC0012021	O=C(c1cccc(c1)C(F)(F)F)Nc1cccc(c1)C(=O)NCC(F)(F)F	-1,10	-4,22	-7,78	-7,58
BCC0077963	O=C(Nc1cccc(c1)C(F)(F)F)CSc1nc(CSc2ccc(cc2)C)cc(=O)[nH]1	8,17	-1,77	-9,43	-16,84
BCC0078313	O=C1N(Cc2nc3ccccc3c(=O)n2Cc2ccco2)C(=O)c2c1cccc2	9,22	10,08	9,22	-3,05
BCC0103586	COc1cc(OCc2ccccc2)ccc1CN1CCN(CC1)C	7,18	7,68	-0,66	-6,97
BCC0024436	Fc1ccc2c(c1)sc(n2)NC(=O)c1noc(c1)C	4,45	0,97	-3,66	-6,56
BCC0038765	N#CC1=C(N)N(c2cccc(c2)[N+](=O)[O-])C2=C(C1c1cnn(c1C)C)C(=O)CCC2	12,80	7,18	-1,67	-3,87

Compound Id	SMILES	receptor-ligand BRET decrease (%) Replicate1	receptor-ligand BRET decrease (%) Replicate2	plasma membrane-ligand BRET decrease (%) Replicate1	plasma membrane-ligand BRET decrease (%) Replicate2
BCC0055213	O=C(Nc1ccc(c(c1)C)C)CCN1CC(C)OC(C1)C	5,80	7,26	-6,10	-4,42
BCC0046681	Brc1ccc(cc1)NC(=O)C1Cc2ccccc2CN1S(=O)(=O)c1cccs1	4,76	1,84	-8,52	-13,41
BCC0088257	CCc1cccc(c1NC(=O)c1ccc(cc1)OC(C)C)C	5,75	2,77	-8,49	-14,18
BCC0083653	O=C(c1ccco1)N1CCN(CC1)c1ccccn1	4,49	5,80	-16,90	-9,88
BCC0047291	O=C(N1CCCc2c1cccc2)CCCn1c(=O)c2cccc3c2c(c1=O)ccc3	7,68	7,27	-6,47	-6,80
BCC0101520	O=C(CSc1ccncc1)NCCc1cc2n(n1)ccc(n2)c1ccncc1	10,08	10,30	-5,62	-3,41
BCC0015005	O=C(NC(C(=O)NCc1ccc2c(c1)OCO2)C)OCc1ccccc1	9,87	8,58	-5,96	-4,66
BCC0121221	O=C1CCC(=NN1c1ccccc1)C(=O)NNC(=O)c1cccs1	7,11	5,29	-9,50	-5,67
BCC0029788	CSc1scc2c1c1n[nH]cc1CC2	9,94	7,79	-7,34	-3,35
BCC0027257	CC(c1ccccc1)NS(=O)(=O)c1ccccc1F	7,92	6,88	-3,95	-6,00
BCC0111722	O=C(Nc1nnc(s1)COc1ccccc1)COc1ccccc1C	6,09	7,50	-7,26	-10,21
BCC0044387	Brc1ccc2c(c1)c(nc(n2)O)c1ccccc1	5,68	6,67	-12,85	-12,58
BCC0005140	O=C(Nc1onc(c1)C(C)C)CSc1ncnc2c1scc2	6,02	1,33	-16,37	-10,97
BCC0108839	O=C(N1c2ccccc2CCc2c1cccc2)CSc1nnnn1c1cccc(c1)C(=O)C	6,53	8,13	-17,03	-3,69
BCC0007587	Fc1ccc(cc1)c1cnc(o1)c1ccccc1C(=O)Nc1ccccc1O	4,47	8,11	-1,08	-5,25
BCC0122740	O=C1Nc2ccccc2N(C1)C(=O)C(Sc1ncnc2c1scc2)C	8,14	7,70	-7,78	-9,59
BCC0032181	Clc1ccc(cc1)n1nnc(c1)C(=O)c1cccs1	9,53	6,39	-6,70	-8,09
BCC0111159	CSCCc1[nH]c(nc1C)SCC(=O)Nc1cccc2c1cccc2	7,74	8,04	-8,85	-7,63
BCC0109120	CC(C(=O)N1c2ccccc2CCc2c1cccc2)n1nnc(n1)c1cccs1	2,37	6,52	-7,27	-4,92
BCC0114565	COc1ccc(cc1)S(=O)(=O)N1CCCC(C1)C(=O)Nc1cccc(c1Cl)Cl	4,71	2,66	-4,12	-6,03
BCC0031632	O/N=C(/CN1CCc2c(C1c1cc(OC)c(c(c1)OC)OC)cc(c(c2)OC)OC)\N	0,27	4,73	-7,34	-7,36
BCC0122352	COc1c(OC)cc(cc1OC)c1nnc(o1)Cn1cc(ccc1=O)C(F)(F)F	2,60	1,47	-8,25	-12,86
BCC0035750	O=C(CC12CC3CC(C2)CC(C1)C3)N/N=C/c1ccc(o1)COc1ccc(cc1)Br	0,88	1,68	-15,81	-14,61
BCC0055139	COc1cc(ccc1OC)C(=O)N1CC(=O)N(C(=O)C1)CCc1ccccc1	1,42	4,36	2,85	-5,33
BCC0112285	O=C(N1CCN(CC1)c1ccccc1F)CCN1C(=O)c2c(C1=O)cccc2	5,26	4,90	-8,21	-1,62
BCC0013320	O=C(c1ccc(cc1)Cn1cccn1)NCc1ccc(cc1)CN1CCCC1	-2,27	-3,67	1,61	-5,27
BCC0122515	Cc1ccc(c(c1)Cl)NC(=O)c1cccc(c1)Br	0,65	-0,39	-1,14	-7,83
BCC0053509	CCC(NC(=O)CCS(=O)(=O)c1cc(Br)cc2c1N(C(=O)C)C(C2)C)C	-1,67	0,96	-4,21	-11,28
BCC0037456	Cc1ncc(nc1)C(=O)N1CCN(CC1)c1ccc(cn1)C(F)(F)F	-0,38	-0,97	-10,04	-13,11
BCC0004615	Fc1ccc(cc1)CNC(=O)c1onc(c1)C	0,70	-5,49	-12,91	-13,93
BCC0018468	COc1ccc(cc1)CCNCc1ccc(o1)C	-5,27	-3,60	-6,67	5,75
BCC0065386	C=CCOc1ccc2c(c1)c(C(=O)OCC)c([nH]2)C	-0,06	2,30	4,81	1,12
BCC0004338	COC(=O)c1ccccc1NC(=O)CSc1nc(C)c(c(n1)C)C	-2,40	-0,11	-7,30	0,72
BCC0000358	O=C(Nc1ccc(c(c1)S(=O)(=O)N(C)C)C)CNc1cc(F)cc(c1)F	2,58	2,58	-2,59	-2,81
BCC0109158	COc1cccc(c1)OCc1nnc(o1)SCC(=O)c1cc(F)ccc1O	2,41	4,35	-5,38	-1,55
BCC0105222	O=C(NCc1ccc2c(c1)OCO2)CSc1nc(N)c(c(=O)[nH]1)c1ccccc1	-2,67	-2,02	-4,66	-18,09
BCC0117498	CNC(=O)CNC(=O)Nc1cccc(c1)C	-2,87	-2,15	-13,27	-15,51
BCC0024921	Fc1cccc(c1)C(=O)N1CCN(CC1)c1ccccc1O	-3,94	-4,51	-15,63	-11,83
BCC0103399	Cc1ccc2c(c1)nc(o2)c1ccc(c(c1)NC(=O)c1ccccc1Br)Cl	-7,46	-0,97	-12,82	-7,74
BCC0017503	Fc1ccc(cc1)c1nn(cc1CNc1ncnc2c1cnn2C)c1ccccc1	-3,55	-6,50	-15,42	-11,15
BCC0099739	COc1ccc2c(c1)OC1(NC2=O)CC2CCC1CC2C(=O)NCc1cccc(c1)F	-1,13	-0,57	-4,67	-8,29
BCC0020406	Cc1ccnc(n1)SCc1nc2n(c1Br)cccc2	-0,42	1,09	-10,35	-12,02
BCC0068132	CN1CCCCC1C#CC(c1ccccc1)(c1ccccc1)O	1,11	3,15	-4,21	-5,19
BCC0003661	O=C(C1CC1)Nc1ccc(cc1)C(=O)NCc1c(C)cc([nH]c1=O)C	1,24	1,21	-7,67	-9,71
BCC0115758	O=C1Nc2ccccc2SC1N1CCCCC1	1,96	2,36	-13,04	-6,03
BCC0017918	COc1ccc2c(c1)sc(n2)NC(=O)c1nccc(c1)Cl	-8,16	-5,12	-9,57	-7,99
BCC0098939	COc1ccc(cc1)S(=O)(=O)N1CCN(CC1)Cc1ccnc(n1)N1CCN(CC1)C	-5,63	-6,38	-7,36	-5,75
BCC0076811	Clc1ccc(cc1)S(=O)(=O)N1CCCC(C1)C(F)(F)F	-3,76	-5,95	-12,08	-8,49
BCC0030558	O=C(c1ccccc1C(=O)c1cc(cc(c1)C(F)(F)F)C(F)(F)F)NCc1ccccn1	-4,24	-4,25	-11,70	-8,89
BCC0018290	COCCNc1nc2ccccc2nc1C(=O)OCC	-4,62	-4,96	-11,63	-19,20
BCC0100640	Fc1ccc(cc1)C1(O)CCC2C1CN(C2)Cc1ccnc(n1)N1CCSCC1	-12,15	-12,84	-11,07	-4,85
BCC0047759	O=C(Nc1cccc(c1)C(=O)C)CNS(=O)(=O)c1c(C)cc(cc1C)C	-0,92	-1,83	5,58	16,48
BCC0028368	Clc1ccc(cc1)Oc1c(C)[nH]nc1c1ccc(cc1O)O	-0,70	2,38	3,73	-5,41
BCC0033957	CN(/C=N/c1cnc2c(c1)c(=O)[nH]c(=O)n2C)C	1,00	-1,95	-5,47	-3,24
BCC0100684	O=C(c1n[nH]c2c1CCC2)N1CCCC2(C1)NC(=O)c1c(O2)cccc1	-0,46	-3,26	-5,70	-9,32
BCC0002702	O=C(c1c(sc(c1C)c1ccccc1)NC(=O)c1ccco1)N1CCOCC1	-1,34	-4,10	-12,16	-14,98

Compound Id	SMILES	receptor-ligand BRET decrease (%) Replicate1	receptor-ligand BRET decrease (%) Replicate2	plasma membrane-ligand BRET decrease (%) Replicate1	plasma membrane-ligand BRET decrease (%) Replicate2
BCC0117062	Oc1cccc(c1)/C=C/c1ccc2c(n1)c(O)ccc2	-3,98	-5,28	-13,27	-14,20
BCC0006634	COc1ccccc1/N=C\1/SC(C(=O)N1C)CC(=O)Nc1ccc(cc1)C(=O)O	-1,40	-4,16	10,21	3,17
BCC0052944	COc1ccc(cc1)NC(=O)CCCn1c(=O)c2sccc2n(c1=O)Cc1ccc(cc1C)C	-1,89	-0,36	0,37	3,69
BCC0106984	Cc1cccc(c1)OCC(=O)Nc1nc2c(s1)cccc2	-1,68	-2,49	-1,94	2,33
BCC0076826	O=C(CN(S(=O)(=O)c1ccccc1)Cc1ccc(cc1)F)Nc1c(C)cccc1C	-1,17	0,20	4,53	-7,57
BCC0122085	COc1cc(cc(c1Br)OC)C(=O)Nn1cn[nH]c1=S	-5,01	-1,17	-0,75	-6,15
**BCC0079473**	O=C(Nc1ccc2c(c1)OCCO2)NCCN1CCN(CC1)c1ccccc1	-54,87	-60,47	-60,52	-57,48
BCC0027747	COc1cc2CN(CCc2cc1OC)C(=O)C1CCN(CC1)C(=O)c1cccs1	-0,88	-4,98	-6,52	-11,96
BCC0121243	O=C(Nn1c(COc2c(C)cccc2C)n[nH]c1=S)Cc1ccccc1	-2,46	-2,36	-1,08	-11,01
BCC0011450	O=C(Cn1ncc(=O)c2c1cccc2)Nc1sc2c(c1C(=O)N)CCC2	-5,78	-4,52	-11,34	-12,05
BCC0110875	Cc1ccc(cc1)N(C(=O)c1cccc(c1)S(=O)(=O)N1CCOCC1)Cc1ccco1	-5,67	-7,01	-14,15	-16,59
BCC0048917	Fc1cccc(c1)CSc1nnc(n1c1ccccc1)C(c1ccccc1)O	-3,22	-1,93	4,66	7,53
Control compounds:					
A-61603	CS(=O)(=O)NC1=C(C=CC2=C1CCCC2C3=NCCN3)O	-72,71	-72,60	-73,97	-76,04
Carvedilol	COC1=CC=CC=C1OCCNCC(COC2=CC=CC3=C2C4=CC=CC=C4N3)O	-99,98	-96,68	-106,26	-107,30
Oxymetazoline	CC1=CC(=C(C(=C1CC2=NCCN2)C)O)C(C)(C)C	-83,78	-77,63	-83,53	-77,74
Prazosin	COC1=C(C=C2C(=C1)C(=NC(=N2)N3CCN(CC3)C(=O)C4=CC=CO4)N)OC	-96,10	-97,24	-100,72	-101,49
DMSO	CS(=O)C	-0,79	0,79	1,98	-1,98

Table S2. Summary of curve fitting results for the main figures

Setup	Constructs expressed	Tracer ligand	Examined compound	Curve fitted	Parameter fitted	Figure
receptor–ligand BRET	NanoLuc–AT_1R, DN-Dyn	TAMRA–AngII (1 µM)	candesartan	Two sites competition binding	IC_{50_Hi} = 520 pM, IC_{50_Lo} = 1 nM	Fig. 2B
receptor–ligand BRET	GLuc–AT_1R, DN-Dyn	TAMRA–AngII (1 µM)	candesartan	Two sites competition binding	IC_{50_Hi} = 207 pM, IC_{50_Lo} = 1.3 nM	Fig. 2C
receptor–ligand BRET	GLuc–AT_1R, DN-Dyn	TAMRA–AngII	TAMRA–AngII	Two sites specific binding	K_{D_Hi} = 1.016 µM K_{D_Lo} = 26.42 µM	Fig. 2D
receptor–ligand BRET	GLuc–α_{1A}AR, DN-Dyn	BODIPY FL–prazosin	BODIPY FL–prazosin	One site specific binding	K_D = 61.13 nM	Fig. 2E
receptor–ligand BRET	GLuc–α_{1A}AR, DN-Dyn	BODIPY FL–prazosin (150 nM)	A61603	Two sites competition binding	K_{i_Hi} = 32.5 pM, K_{i_Lo} = 187 nM	Fig. 2F
receptor–ligand BRET	GLuc–α_{1A}AR, DN-Dyn	BODIPY FL–prazosin (150 nM)	prazosin	One site competition binding	K_i = 4.17 nM	Fig. 2F
receptor–ligand BRET	GLuc–α_{1A}AR, DN-Dyn	BODIPY FL–prazosin (150 nM)	carvedilol	One site competition binding	K_i = 62.7 nM	Fig. 2F
plasma membrane–ligand BRET	NanoLuc–PM, AT_1R, DN-Dyn	TAMRA–AngII (1 µM)	candesartan	Two sites competition binding	IC_{50_Hi} = 1.29 nM, IC_{50_Lo} = 19.6 nM	Fig. 2H
plasma membrane–ligand BRET	GLuc–PM, AT_1R, DN-Dyn	TAMRA–AngII (1 µM)	candesartan	Two sites competition binding	IC_{50_Hi} = 1.24 nM, IC_{50_Lo} = 248 µM	Fig. 2I
plasma membrane–ligand BRET	GLuc–PM, AT_1R, DN-Dyn	TAMRA–AngII	TAMRA–AngII	Two sites specific binding	K_{D_Hi} = 287 nM, K_{D_Lo} = 18.46 µM	Fig. 2L
plasma membrane–ligand BRET	GLuc–PM, AT_1R, DN-Dyn	TAMRA–AngII (1 µM)	AngII	Two sites competition binding	IC_{50_Hi} = 105 nM, IC_{50_Lo} ambiguous	Fig. 2M
plasma membrane–ligand BRET	GLuc–PM, AT_1R, DN-Dyn	TAMRA–AngII (1 µM)	SII	Two sites competition binding	IC_{50_Hi} = 1.28 µM, IC_{50_Lo} ambiguous	Fig. 2M
plasma membrane–ligand BRET	GLuc–PM, α_{1A}AR, DN-Dyn	BODIPY FL–prazosin	BODIPY FL–prazosin	One site specific binding	K_D = 46.63 nM	Fig. 2N
plasma membrane–ligand BRET	GLuc–PM, α_{1A}AR, DN-Dyn	BODIPY FL–prazosin (150 nM)	A61603	Two sites competition binding	K_{i_Hi} = 94.8 pM, K_{i_Lo} = 348.8 nM	Fig. 2O
plasma membrane–ligand BRET	GLuc–PM, α_{1A}AR, DN-Dyn	BODIPY FL–prazosin (150 nM)	prazosin	One site competition binding	K_i = 2.72 nM	Fig. 2O
plasma membrane–ligand BRET	GLuc–PM, α_{1A}AR, DN-Dyn	BODIPY FL–prazosin (150 nM)	carvedilol	One site competition binding	K_i = 43.8 nM	Fig. 2O
plasma membrane–ligand BRET	GLuc–PM, β_2AR, DN-Dyn	BODIPY FL–(S)-propranolol (300 nM)	ICI118,551	One site competition binding	IC_{50} = 9.84 nM	Fig. 3A
plasma membrane–ligand BRET	GLuc–PM, D_1R, DN-Dyn	BODIPY FL–SKF83566 (300 nM)	SCH 23390	One site competition binding	IC_{50} = 1.07 nM	Fig. 3B
plasma membrane–ligand BRET	GLuc–PM, AT_1R, DN-Dyn	TAMRA–AngII (1 µM)	AngII	One site competition binding	IC_{50} = 479 nM	Fig. 3C
plasma membrane–ligand BRET	GLuc–PM, EGFR, DN-Dyn	Alexa488–EGF (300 ng/ml)	EGF	One site competition binding	IC_{50} = 15.8 pg/l	Fig. 3D
plasma membrane–ligand BRET	GLuc–PM, TfR–HA, DN-Dyn	Alexa488–transferrin (100 µg/ml)	transferrin	One site competition binding	IC_{50} = 4.54 µg/l	Fig. 3E
receptor–ligand BRET	GLuc–α_{1A}AR, DN-Dyn	BODIPY FL–prazosin (150 nM)	BODIPY FL–prazosin	Association kinetics - one conc. of hot	$k_{on} = 3.86\times10^4\ s^{-1}M^{-1}$	Fig. 4B
receptor–ligand BRET	GLuc–α_{1A}AR, DN-Dyn	BODIPY FL–prazosin (150 nM)	BODIPY FL–prazosin	One phase exponential decay	$k_{off} = 1.84\times10^{-3}\ s^{-1}$	Fig. 4A
receptor–ligand BRET	GLuc–α_{1A}AR, DN-Dyn	BODIPY FL–prazosin (150 nM)	prazosin	Kinetics of competitive binding	$k_{on} = 2.38\times10^6\ s^{-1}M^{-1}$	Fig. 4B
receptor–ligand BRET	GLuc–α_{1A}AR, DN-Dyn	BODIPY FL–prazosin (150 nM)	prazosin	Kinetics of competitive binding	$k_{off} = 3.51\times10^{-3}\ s^{-1}$	Fig. 4B
plasma membrane–ligand BRET	GLuc–PM, α_{1A}AR, DN-Dyn	BODIPY FL–prazosin (150 nM)	BODIPY FL–prazosin	Association kinetics - one conc. of hot	$k_{on} = 3.22\times10^4\ s^{-1}M^{-1}$	Fig. 4B
plasma membrane–ligand BRET	GLuc–PM, α_{1A}AR, DN-Dyn	BODIPY FL–prazosin (150 nM)	BODIPY FL–prazosin	One phase exponential decay	$k_{off} = 1.33\times10^{-3}\ s^{-1}$	Fig. 4A
plasma membrane–ligand BRET	GLuc–PM, α_{1A}AR, DN-Dyn	BODIPY FL–prazosin (150 nM)	prazosin	Kinetics of competitive binding	$k_{on} = 1.7\times10^6\ s^{-1}M^{-1}$	Fig. 4B
plasma membrane–ligand BRET	GLuc–PM, α_{1A}AR, DN-Dyn	BODIPY FL–prazosin (150 nM)	prazosin	Kinetics of competitive binding	$k_{off} = 2.55\times10^{-3}\ s^{-1}$	Fig. 4B
receptor–ligand BRET	GLuc–AT_1R, DN-Dyn	TAMRA–AngII (1 µM)	TAMRA–AngII	Association kinetics - one conc. of hot	$k_{on} = 1.15\times10^4\ s^{-1}M^{-1}$	Fig. 4D
receptor–ligand BRET	GLuc–AT_1R, DN-Dyn	TAMRA–AngII (1 µM)	TAMRA–AngII	One phase exponential decay	$k_{off} = 4.05\times10^{-2}\ s^{-1}$	Fig. 4C
receptor–ligand BRET	GLuc–AT_1R, DN-Dyn	TAMRA–AngII (1 µM)	candesartan	Kinetics of competitive binding	$k_{on} = 5.4\times10^5\ s^{-1}M^{-1}$	Fig. 4E
receptor–ligand BRET	GLuc–AT_1R, DN-Dyn	TAMRA–AngII (1 µM)	candesartan	Kinetics of competitive binding	$k_{off} = 1.23\times10^{-3}\ s^{-1}$	Fig. 4E
plasma membrane–ligand BRET	GLuc–PM, AT_1R, DN-Dyn	TAMRA–AngII (1 µM)	TAMRA–AngII	Association kinetics - one conc. of hot	$k_{on} = 5.27\times10^3\ s^{-1}M^{-1}$	Fig. 4D
plasma membrane–ligand BRET	GLuc–PM, AT_1R, DN-Dyn	TAMRA–AngII (1 µM)	TAMRA–AngII	One phase exponential decay	$k_{off} = 1.73\times10^{-2}\ s^{-1}$	Fig. 4C
plasma membrane–ligand BRET	GLuc–PM, AT_1R, DN-Dyn	TAMRA–AngII (1 µM)	candesartan	Kinetics of competitive binding	$k_{on} = 2.78\times10^5\ s^{-1}M^{-1}$	Fig. 4E
plasma membrane–ligand BRET	GLuc–PM, AT_1R, DN-Dyn	TAMRA–AngII (1 µM)	candesartan	Kinetics of competitive binding	$k_{off} = 8.33\times10^{-4}\ s^{-1}$	Fig. 4E
plasma membrane–ligand BRET	GLuc–PM, α_{1A}AR, DN-Dyn	BODIPY FL–prazosin (375 nM)	BCC0010913	One site competition binding	IC_{50} = 1.845 µM	Fig. 5C
plasma membrane–ligand BRET	GLuc–PM, α_{1A}AR, DN-Dyn	BODIPY FL–prazosin (375 nM)	BCC0079473	One site competition binding	IC_{50} = 121.6 nM	Fig. 5C
plasma membrane–ligand BRET	GLuc–PM, α_{1A}AR, DN-Dyn	BODIPY FL–prazosin (375 nM)	BCC0072526	One site competition binding	IC_{50} = 86.12 nM	Fig. 5C
plasma membrane–ligand BRET	GLuc–PM, α_{1A}AR, DN-Dyn	BODIPY FL–prazosin (375 nM)	BCC0061678	One site competition binding	IC_{50} = 180.4 nM	Fig. 5C
